# Neuronal and behavioral affective perceptions of human and naturalness-reduced emotional prosodies

**DOI:** 10.3389/fncom.2022.1022787

**Published:** 2022-11-18

**Authors:** Mathilde Marie Duville, Luz María Alonso-Valerdi, David I. Ibarra-Zarate

**Affiliations:** Tecnologico de Monterrey, Escuela de Ingeniería y Ciencias, Monterrey, NL, Mexico

**Keywords:** electroencephalography (EEG), single-trial event-related potential (ERP), affective prosody, emotions, naturalness, valence, arousal, synthesized speech

## Abstract

Artificial voices are nowadays embedded into our daily lives with latest neural voices approaching human voice consistency (naturalness). Nevertheless, behavioral, and neuronal correlates of the perception of less naturalistic emotional prosodies are still misunderstood. In this study, we explored the acoustic tendencies that define naturalness from human to synthesized voices. Then, we created naturalness-reduced emotional utterances by acoustic editions of human voices. Finally, we used Event-Related Potentials (ERP) to assess the time dynamics of emotional integration when listening to both human and synthesized voices in a healthy adult sample. Additionally, listeners rated their perceptions for valence, arousal, discrete emotions, naturalness, and intelligibility. Synthesized voices were characterized by less lexical stress (i.e., reduced difference between stressed and unstressed syllables within words) as regards duration and median pitch modulations. Besides, spectral content was attenuated toward lower F2 and F3 frequencies and lower intensities for harmonics 1 and 4. Both psychometric and neuronal correlates were sensitive to naturalness reduction. (1) Naturalness and intelligibility ratings dropped with emotional utterances synthetization, (2) Discrete emotion recognition was impaired as naturalness declined, consistent with P200 and Late Positive Potentials (LPP) being less sensitive to emotional differentiation at lower naturalness, and (3) Relative P200 and LPP amplitudes between prosodies were modulated by synthetization. Nevertheless, (4) Valence and arousal perceptions were preserved at lower naturalness, (5) Valence (arousal) ratings correlated negatively (positively) with Higuchi’s fractal dimension extracted on neuronal data under all naturalness perturbations, (6) Inter-Trial Phase Coherence (ITPC) and standard deviation measurements revealed high inter-individual heterogeneity for emotion perception that is still preserved as naturalness reduces. Notably, partial between-participant synchrony (low ITPC), along with high amplitude dispersion on ERPs at both early and late stages emphasized miscellaneous emotional responses among subjects. In this study, we highlighted for the first time both behavioral and neuronal basis of emotional perception under acoustic naturalness alterations. Partial dependencies between ecological relevance and emotion understanding outlined the modulation but not the annihilation of emotional integration by synthetization.

## Introduction

The last two decades have seen a steady growth in the development of artificial voices with the integration of smart speakers into entertainment, health care, education, marketing, and social sectors. Conversely, healthy populations still perceive synthesized voices as less trustworthy, less pleasant, less likeable and more eerie than human voices (Baird et al., 2018; Kühne et al., 2020). Besides, people tend to be more attentive, engaged, and emotionally responsive, as well as better at retaining information when they interact with human voices (Rodero and Lucas, 2021).

Successful steps were made by roboticists for synthesized speech to acquire clarity (intelligibility) and consistency (naturalness). From concatenative methods such as Pitch Synchronous Overlap-Add (PSOLA) with standard voices, up to Statistical Parametric Speech Synthesis creating neural voices (Ning et al., 2019), the latest synthesized voices (neural voices) have acquired intelligibility close to human voices, but progress still needs to be done to enhance naturalness. When listening to natural speech, the auditory system must encode acoustic information into a biological electric signaling to reach sensorial and cognitive functions necessary for optimized interactions. The efficient neural coding theoretical framework specifies that mammalian perceptual systems evolved to encode environmental stimuli in the most efficient way to promote organisms survival (Zhou and Yu, 2018). It was defined as the minimization of neuronal spikes to transmit information with the highest fidelity at the lowest cost (Zhou and Yu, 2018; Gervain and Geffen, 2019). This way, the auditory system is optimized to integrate spectro-temporal acoustic features and amplitude modulations of naturalistic sounds (Gervain and Geffen, 2019). Therefore, human beings process sounds of ecological relevance, such as human speech, with particular efficiency because of phylogenetic adaptations of sensorial and cognitive systems (Gervain et al., 2016). In sum, the neural specialization of the human brain to process speech is underlined by acoustic properties that are perceived as voice naturalness (i.e., speech intrinsic property to be recognized as a social ecological sound).

Formants (F1, F2, …, Fn), harmonics, and lexical stress patterns were defined to shape voice naturalness. Lexical stress awareness, known as the ability to discern the relative prominence of specific syllables within words, requires the efficient recognition of time and intensity-related acoustic cues that support word isolation, speech understanding (Gutiérrez-Palma et al., 2016) and naturalistic perception of language-specific speech (Schwab and Dellwo, 2017). Speech shows statistical properties of both environmental sounds (consonants) and harmonic vocalizations (vowels), which triggers transient statistical variations of amplitude and spectral structures (Gervain and Geffen, 2019). The neuronal responsivity matches statistical patterns of natural stimuli (e.g., topographic mapping, frequency tuning) (Amin et al., 2013; Zhao et al., 2019), therefore deviations of synthetic voices away from naturalistic statistical models of speech may in part explain the perception of naturalness reduction. Formant frequencies contribute to speech naturalness perception and are particularly useful cues for vowel discrimination, sound subjective preferences and gender attribution (Vos et al., 2018; Zhao et al., 2019; Hardy et al., 2020). Formants, speech rate, and median pitch act together as acoustic cues that help gender naturalistic discrimination in voice. A previous work highlighted that maximum, minimum, and mean pitch frequencies explained 71.2% of the variance for gender recognition in voice, and average frequency over F1, F2, and F3 was a significant predictor of both gender attribution, and naturalness perception (Hardy et al., 2020). Those findings emphasize the complex correlations between the acoustic content and the perception of speech waveforms.

Emotional prosodies are embedded into social ecological representations of speech and key acoustic patterns may serve as predictors for emotional recognition (Aldeneh and Mower Provost, 2021). For instance, Spanish men express joy with higher pitch, speech rate, loudness, and lower harmonics-to-noise ratio than sadness. Mexican children express fear with higher speech rate, pitch, and higher loudness and pitch fluctuations than happiness, while Mexican adult females utter happiness with higher pitch and higher F3 frequency than fear (Duville et al., 2021a). Female Chinese adults tend to express boredom by higher pitch and loudness fluctuations than exuberance (Huang et al., 2021). Although recent advances highlighted the inclusion of emotional prosodic patterns to last created synthesized voices (Xue et al., 2018; Schuller and Schuller, 2021), while most current text-to-speech systems only offer basic prosodic adjustments such as pitch, loudness, and speech rate (e.g., IBM^®^ Watson,^1^ Microsoft^®^ Azure^2^). The oversimplification of prosodic acoustic patterns may trigger slighter emotional responses and naturalistic perceptions than human voices.

Emotional text-to-speech synthesis still needs significant progress for human listeners to be able to correctly categorize emotions (Liu et al., 2021). For now, no text-to-speech service provides discrete emotional categorizations of the synthesized voices they offered. Therefore, we propose to explore the acoustic tendencies of neutral utterances over human, neural, and standard voices to highlight deviations of synthesized voices from the statistical structure of natural speech. Then, we reduced naturalness out of emotional utterances by acoustic edition following tendencies previously highlighted, while conserving emotional prosody. Finally, we assessed behavioral (by using psychometric scales) and neurophysiological perceptions of both human and naturalness-reduced utterances in a healthy adult sample.

The brain’s electrical currents can be recorded over the scalp by electroencephalography (EEG), which is particularly suitable for the inspection of dynamic cortical processes with high temporal resolution. Sensorial and cognitive encodings of emotional prosody may be modeled in three stages through which the listener integrates the acoustic characteristics and the meaning of utterances (Schirmer and Kotz, 2006). Initially, a basic sensory encoding of physical properties (e.g., pitch and loudness) occurs around 100 ms after stimulus onset with a negative peak. N100 modulation by emotionality is controversial and rarely observed for emotional speech processing (Pell et al., 2015; Paulmann and Uskul, 2017) for no modulation observed, and (Pinheiro et al., 2015) for higher negativity for happiness vs. anger. Sensorial analyses are followed by salience appraisal in which emotional cues are integrated. P200, peaking 150–250 ms after stimulus onset over frontal and central electrodes reflects early emotional detection based on relevant acoustic features. Its amplitude is potentiated with salience and motivational significance. Early differentiation of prosodies occurs and the speaker’s arousal (i.e., calm vs. excited) starts to be noticed (Paulmann et al., 2013). Third stage emotional prosody processing is characterized by higher-order cognitive processes for evaluation, interpretation, contextual relevance, mental and memory representations. It is linked to the Late Positive Complex that covers long-lasting Late Positive Potentials (LPP) between 400 and 1,000 ms after stimulus onset over central and parietal cortices. Higher amplitudes refer to more persevering and sustained monitoring of affective information (Pell and Kotz, 2021).

Emotional encoding of prosodies may be further characterized by the geometric complexity of EEG signal temporal sequences. Fractal dimension is a measure of self-similarity that assumes neither linearity nor stationarity in brain signals. The computation of Higuchi’s Fractal Dimension (HFD) associated with emotional information perception allows the differentiation of discrete emotions processing (Ruiz-Padial and Ibáñez-Molina, 2018; Zheng et al., 2021).

An extensive work has been done to detangle the human processing of emotional natural speech, but no research has examined the emotional perception induced by less ecological (i.e., synthesized) voices. Thus, the present investigation was engineered to investigate the encoding of emotional information triggered by human and naturalness-reduced utterances. This study is a step further to the understanding of the cognitive processing of non-human voices.

We schedule to highlight temporal and spectral acoustic deviations from natural speech to synthesized utterances. By further editing them from natural emotional speech, we expect to approximate the reduced naturalness of synthesized voices onto controlled affective stimuli that will be used to assess neuronal and behavioral perceptions of emotions embedded into synthesized speech. We anticipate lower naturalness ratings for the newly created naturalness-reduced voices. Furthermore, if the naturalistic quality of speech, that is, the ecological perception, interferes with emotional apprehensions, then we predict poorer emotional recognition as naturalness reduces. It would be highlighted at a behavioral level by incorrect emotion recognition, and at neuronal level by atypical P200 and LPP amplitude differentiations between prosodies. This is the first study to investigate the electroencephalographic time-course of emotion perception conveyed by synthesized voices, therefore no hypothesis about the direction of P200 an LPP modulation by synthetization could be formulated beforehand.

## Materials and methods

**FIGURE 1:**
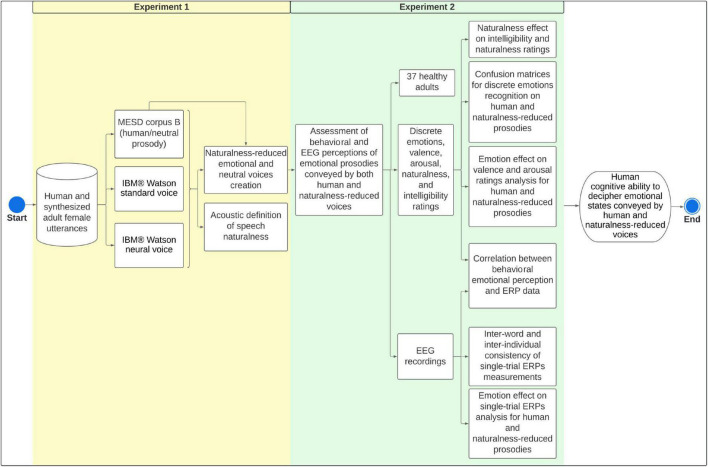
Methodological framework for experiments 1 and 2. We aimed to explore acoustic cues of voice naturalness and create naturalness-reduced synthesized versions of emotional and neutral utterances. Finally, we assessed behavioral and EEG correlates of emotional perception conveyed by both human and naturalness-reduced voices. MESD, Mexican Emotional Speech Database; ERPs, Event-Related Potentials; EEG, Electroencephalography.

Figure 1 summarizes the overall methodological sequence for acoustic definition of naturalness and creation of naturalness-reduced emotional voices (Experiment 1), and assessment of emotions and naturalness perceptions by EEG and psychometric scales (Experiment 2).

### Experiment 1: Naturalness reduction of emotional prosodies

**FIGURE 2:**
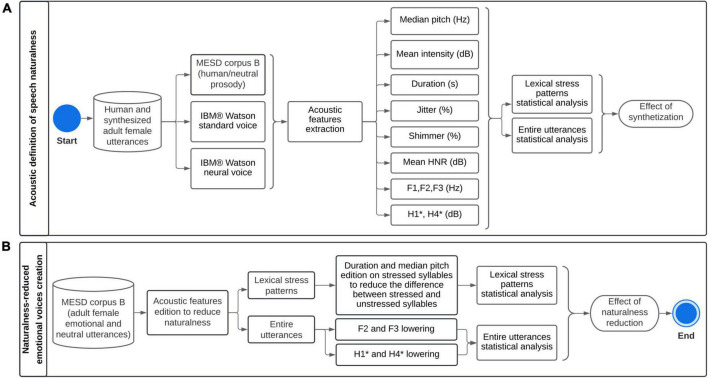
Methodological framework for **(A)** acoustic definition of speech naturalness, and **(B)** creation of naturalness-reduced emotional and neutral utterances. MESD, Mexican Emotional Speech Database; HNR, Mean harmonics-to-noise ratio. F1, F2, and F3: Formants 1, 2 and 3, H1* and H4*: Intensity of harmonics 1 and 2, corrected for formant bias.

Figure 2 summarizes the methodological framework for defining speech naturalness (A) and creating naturalness-reduced emotional and neutral utterances (B).

#### Acoustic definition of speech naturalness

##### Acquisition of human, neural and standard voices

The 24 adult female neutral utterances from the Mexican Emotional Speech Database (MESD) corpus B (Duville et al., 2021a,b) were used for human voices. Synthesized voices (least natural) were acquired by entering the corresponding words in the IBM^®^ Watson text-to-speech service using standard (es-US_SofiaVoice) and neural (es-US_SofiaV3Voice) versions of the female Spanish North American voice Sofia. The Audio Toolbox^3^ from Matlab R2019b was used to interlink with the cloud-based Application Programming Interface. Wav format audio files were written as a sequence of 24-bit with a sample rate of 48,000 Hz.

##### Acoustic features extraction

First, we were interested in exploring lexical stress patterns. EasyAlign Toolkit from Praat (Goldman, 2011; Boersma and Weenink, 2020) was used to perform phonetic segmentation based on the Hidden Markov Model Praat Toolkit. Individual TextGrid files were generated for each word and contained *phone, syllable, and word* tiers resulting from macro-segmentation, grapheme-to-phoneme conversions, and phone segmentation. As EasyAlign is a semi-automatic system, each step was monitored, and manual adjustments were made when necessary. Syllabic units were then extracted and stored in individual wav files as a sequence of 24-bit with a sample rate of 48,000 Hz. Praat was used to extract median pitch, duration, and intensity on stressed and unstressed syllables for lexical patterns analysis. The unstressed syllables of each word were concatenated for mean intensity (dB) and median pitch (Hz) measurements, and mean duration (seconds) of unstressed syllables was considered. Pitch detection was based on the algorithm described by Boersma (1993) which relied on periodicity detection in the autocorrelation domain. Median pitch was measured in Hertz and defined as the 50% quantile. Time step was set at 100 pitch values per second, pitch floor was set at 100 Hz and pitch ceiling at 600 Hz.

Second, prosodic, voice quality, and spectral tendencies were analyzed on entire utterances. Praat was used to extract mean intensity, jitter local, jitter ppq5, shimmer local, shimmer ppq5, mean harmonics-to-noise ratio (HNR), and F1 to F3 frequencies. A description of those features is detailed in Supplementary File 1 (Supplementary Table 1) (Liu et al., 2018; Akçay and Oğuz, 2020; Singh et al., 2021). Before extracting mean intensity, the amplitude of acoustic waveforms was rescaled between −1 and 1, following Equation (1).


(1) $$X_{normalized}=\frac{x}{\text{max}\text{[}abs_{j}\left(X\right)]}$$

where *x* is the value to be normalized, and *max*[*abs*_j_(X)] is the highest value of the absolute waveform.

Thereafter, Matlab R2019b was used to compute the intensity of the 1st (fundamental frequency F0), 2nd, 3rd, and 4th harmonics (H1, H2, H3, and H4) on normalized acoustic waveforms. First, the Power Spectral Density (PSD) was estimated by computing a modified periodogram with a Hamming window. The number of discrete Fourier Transform (DFT) points was determined by Equation (2).


(2) $$NumberofDFTpoints=2^{n}$$

where *n* is the nearest decimal integer of *log*_2_(*waveformnumberofsamples*).

Then, intensities expressed in dB were corrected for formant bias, according to the formula correction proposed by Iseli and Alwan (2004) and described in Supplementary File 1 (Supplementary Table 1). Specifically, H1, H2, H3, and H4 were corrected for the effect of formants 1 and 2. Corrected harmonics are named H1*, H2*, H3*, and H4*.

##### Statistical analysis

Statistical analysis was performed with R software (R Foundation for Statistical Computing, Vienna, Austria). Level of significance was set at *p* < 0.05.

For lexical stress patterns analysis, differences between stressed and unstressed syllables were computed (stressed minus unstressed). A one-way repeated measures ANOVA with type of voice as factor (human, standard, and neural) was conducted on the difference variable for each acoustic feature independently. Mauchly’s test of sphericity was used to evaluate homogeneity of variances and co-variances. In case of violation of sphericity, a Greenhouse-Geisser correction was conducted. Normality of residuals was assessed with Shapiro-Wilk test. In case of non-normal distribution, Friedman test was used. *Post-hoc* comparisons were conducted to assess specific differences (Tukey after ANOVA, Conover with *p*-value adjustment by Holm method after Friedman).

Then, adult female emotional and neutral utterances (anger, disgust, fear, happiness, neutral, and sadness) from the MESD corpus B were used to explore tendencies from unstressed to stressed syllables in the human emotional voice. The 24 available utterances were used for every emotion. Paired *t*-tests were conducted on each acoustic feature separately. Normality of differences was assessed with Shapiro-Wilk test. In case of non-parametric distribution, paired Wilcoxon tests were applied.

Finally, to explore the effect of synthetization on entire utterances, values from each acoustic feature were rescaled between 0 and 1 to reduce inter-individual biases according to the min-max normalization as described in Equation (3).


(3) $$x_{normalized}=\frac{x-min_{k}}{max_{k}-min_{k}}$$

where *x* is the value to be normalized, *max*_*k*_ is the highest value of acoustic feature *k* and *min*_*k*_ is the lowest value of *k*.

A one-way repeated measures ANOVA with type of voice as factor was conducted on normalized values of each acoustic feature separately. Mauchly’s test of sphericity was used to evaluate homogeneity of variances and co-variances. In case of violation of sphericity, a Greenhouse-Geisser correction was conducted. Normality of residuals was assessed with Shapiro-Wilk test. In case of non-normal distribution, Friedman test was used. *Post hoc* comparisons were conducted to assess specific differences (Tukey after ANOVA, Conover with *p*-value adjustment by Holm method after Friedman).

#### Naturalness-reduced emotional voices creation

##### Acoustic edition of human voice

Naturalness was progressively reduced from human voice to level 2, creating three levels of naturalness under study (human, level 1, and level 2). Acoustic features that were previously highlighted to gradually increase or decrease from human to neural to standard voices were edited from MESD utterances. The 24 utterances per emotion originally present in MESD were considered in every level. To avoid a perfect linear correlational fit between levels and to guarantee the reliability of further statistical analysis of variances (McDonald, 2014), the degree of naturalness varied within single levels and reduction was non-equidistant across levels, leading to 38% (SD = 15%) and 74% (SD = 18%) reduction for levels 1 and 2, respectively, based on the human voice.

Duration and median pitch were edited on stressed syllables to reduce the difference between stressed and unstressed syllables. The Vocal Toolkit from Praat software^4^ was used to apply the time domain PSOLA method. Particularly, speech fragments from stressed syllables were windowed by a Hanning window centered at pitch periods with 50% overlapping. Pitch and duration were either decreased or increased to reduce the difference from unstressed syllables. Pitch decrease was reached by reducing window overlap length, triggering longer periods and lowering F0. The opposite (i.e., increasing overlap length) was done to increase pitch. Duration decrease was reached by cutting windowed segments out of the acoustic waveform, whereas duration increase involved the duplication of windowed segments (Moulines and Charpentier, 1990). This technique did not alter other parameters than F0 and duration as it simply copied or cut segments from the original signal, so that vocal tract filter properties stayed intact.

Then, Matlab R2019b was used to concatenate the edited stressed and the unstressed syllables to recompute individual words. As abrupt variations of pitch and duration can cause the psychoacoustic perception of an additional sound coming from a new source (i.e., the superposition of an external sound on the voice) (Moore, 2007), speech segments were cross-faded over a 10 ms window. Namely, the cross-faced window included the last 5 ms of the first syllable, and first 5 ms of the second syllable. The fading curves were generated by a linearly spaced vector from 0 to 1 (fade in) or 1 to 0 (fade out), for which the spacing between the points followed the mathematical formula described in Equation (4). This procedure ensured a smoothed transition between edited and unedited syllables.


(4) $$\frac{x_{2}-x_{1}}{n-1}$$

where *x_2_* was set to 0 in case of fade in and 1 in case of fade out, *x_1_* was set to 1 in case of fade in, and 0 in case of fade out, and *n* was equal to the number of samples of the segment to be faded.

At that point, the Vocal Toolkit from Praat was used to edit F2 and F3 frequencies of concatenated speech utterances from levels 1 and 2 as defined in Table 1. Specifically, the hierarchy between resonance frequencies had to be preserved, so that F1 was lower than F2, which was lower than F3. As a result, level 1 was characterized by a 12% reduction with 7% standard deviation, and level 2 by a 41% reduction with a standard deviation of 7%.

**TABLE 1:** Ratios between formants for human voice, level 1, and level 2.

	F2/F1 human	F3/F1 human	F2/F1 level 1	F3/F1 level 1	F2/F1 level 2	F3/F1 level 2
Mean	2.8	5.1	2.5	4.4	1.7	3.0
SD	0.7	0.8	0.7	0.8	0.5	0.6

Note that non-equidistant ratios between levels were defined to avoid a perfect linear correlational fit and guarantee the reliability of further statistical analysis of variances.

Then, the Audio Toolbox from Matlab R2019b was used to perform multiband parametric equalization to reduce the intensity of harmonics 1 and 4. Harmonics intensity and center frequencies were calculated by computing the PSD. The bandwidths that defined the equalizer were the harmonics bandwidths. They were computed as the distance between the points where the descending signal intercepted a horizontal reference line positioned beneath the peak at a vertical distance equal to half the peak prominence.

##### Statistical analysis

At each edition step, paired *t*-tests were conducted to compare adjusted acoustic parameters with those theoretically expected. Normality of differences was assessed with Shapiro-Wilk test. In case of non-parametric distribution, paired Wilcoxon tests were applied.

After all editions were made, acoustic waveforms from emotional and neutral utterances of human voice, level 1 and 2 were rescaled between −1 and 1 according to Equation (1), and levels comparisons were conducted. A one-way repeated measures ANOVA with naturalness as factor was applied on each acoustic feature independently (F2, F3, H1*, H4*, difference variable for median pitch and duration for lexical stress). Mauchly’s test of sphericity was used to evaluate homogeneity of variances and co-variances. In case of violation of sphericity, a Greenhouse-Geisser correction was conducted. Normality of residuals was assessed with Shapiro-Wilk test. In case of non-normal distribution, Friedman test was used. *Post-hoc* comparisons were conducted to assess specific differences (Tukey after ANOVA, Conover with *p*-value adjustment by Holm method after Friedman).

### Experiment 2: Electroencephalographic response to human and naturalness-reduced voices

#### Participants

37 healthy adults were recruited for this study [16 females, mean age; SD; range = 25.81; 4.33; (19–35)]. Participants had no history of language, cognitive, hearing, psychiatric, or psychologic pathology. They all had normal or corrected-to-normal vision. No participant was under medication affecting central or peripheral nervous system at the time of the study. All participants were Mexican, currently living in Mexico, with Spanish as their mother-tongue, brought up in Mexican families and with a Mexican academic education.

#### Experimental procedure

**FIGURE 3:**
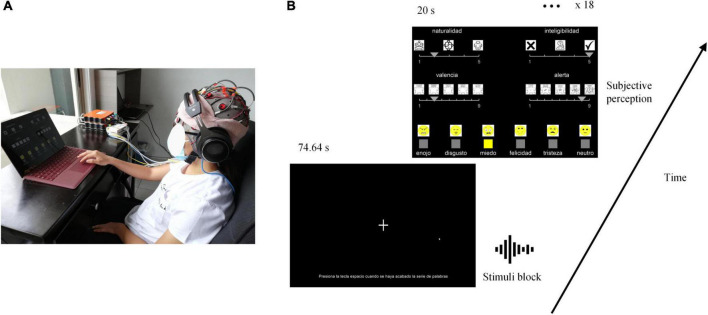
Experimental set-up. Experimental conditions are presented in **(A)**, and the task timeline in **(B)**. “naturalidad (sp)” is naturalness (en), “inteligibilidad” is intelligibility, “valencia” is valence, “alerta” is arousal, “enojo” is anger, “disgusto” is disgust, “felicidad” is happiness, “tristeza” is sadness, “neutro” is neutral.

Auditory stimuli were the 432 single-word emotional utterances, corresponding to 144 utterances per level of naturalness (i.e., 24 utterances per emotion in each level of naturalness previously created). Stimuli were displayed at 60 dB via the Shure SRH1840 audio headset that has a flat frequency response to accurately reproduce the input audio signal. Participants were seated comfortably in an armchair in front of a computer screen while their EEG activity was recorded as can be seen in Figure 3A.

Instructions were explained both verbally and in writing on the computer screen, and participants were told to ask all questions needed before starting the session. At the beginning of the experiment, participants were asked to relax for 60 s, and get prepared to focus on the task.

As illustrated in Figure 3B, stimuli were presented by blocks of 24 words, corresponding to one level of naturalness and one emotion. In each block, stimuli were presented consecutively with a 3.11 s stimulus-onset asynchrony. The stimulus sequence for each block and the order of blocks were randomized. After each block, participants were asked to evaluate naturalness, intelligibility, valence, arousal, and qualitative emotion. The Mean Opinion Score was used to score naturalness and intelligibility using a 5-point scale (respectively: 1 = unnatural/artificial, 5 = natural/human, and 1 = not much easy to understand, 5 = easy to understand) (Viswanathan and Viswanathan, 2005; Tamura et al., 2015; Ramu Reddy and Sreenivasa Rao, 2016). Valence and arousal were scored using the Self-Assessment Manikin Scales (Bradley and Lang, 1994; Gatti et al., 2018). Qualitative emotions were evaluated by choosing between anger, disgust, fear, happiness, neutral, and sadness. Each scale was illustrated by icons to facilitate the understanding and process. Icons for qualitative emotions were the same as in Gao et al. (2014). The positions of scales on the screen were randomly distributed and counterbalanced between participants. Participants were given 20 s to rate all dimensions. The graphical user interface provided to the participant and the sequence of the task is illustrated in Figure 3B. PsychoPy3 (3.2.4) (Peirce et al., 2019, p. 2) was used to generate the graphical user interface and gather the subjective ratings. OpenVibe (1.3.0) (Renard et al., 2010) was used to design the auditory paradigm and register EEG recordings.

#### Event-related potentials recording and processing

**FIGURE 4:**
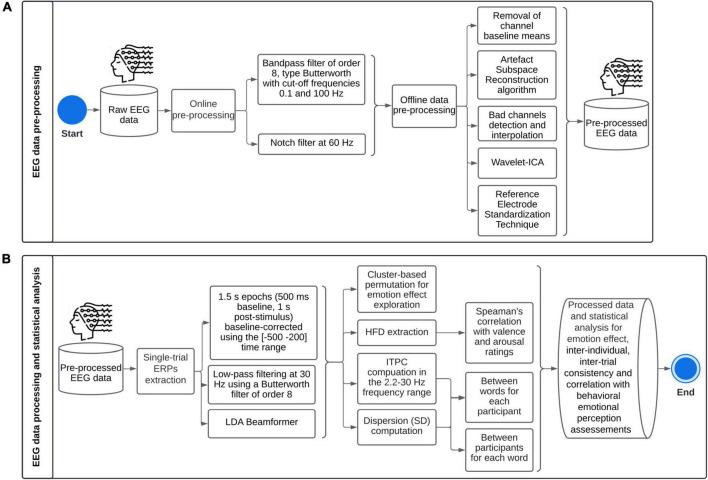
Methodological framework for single-trial Event-Related Potentials: **(A)** pre-processing, **(B)** processing and statistical analysis. ICA, Independent Components Analysis; ERPs, Event-Related Potentials; LDA, Linear Discriminant Analysis; HFD, Higuchi’s fractal dimension; ITPC, Inter-Trial Phase Coherence; SD, Standard Deviation.

Continuous EEG data was acquired from a 32-channel EEG amplifier system (gUSBamp, gTec) with Ag/AgCl scalp electrodes placed according to the international 10–20 system on GAMMAcap3 headset and a 256 Hz sampling rate. Electrodes included: Fp1, Fp2, AF3, AF4, F7, F3, Fz, F4, F8, FC5, FC1, FC2, FC6, T7, C3, Cz, C4, T8, CP5, CP1, CP2, CP6, P7, P3, Pz, P4, P8, PO7, PO3, PO4, PO8, Oz. During online recording, AFz was used as ground, and data was referenced to the left earlobe. Electrode impedance was kept below 5 kΩ. Figure 4 summarizes EEG data pre-processing, processing, and statistical analysis.

EEGLab toolbox version 2021.0 from Matlab was used to pre-process and process the data. High variance spontaneous artifacts were removed by the Artifact Subspace Reconstruction algorithm (Chang et al., 2020). Bad channels were depicted as (1) having a flatline longer than 5 s, or (2) presenting more line noise relative to its signal than 4 standard deviations based on the total channel population, or (3) channels which joint log probability fell more than 5 standard deviations from the mean of the probability density function of the whole channel population (Delorme et al., 2007); and were then interpolated by the superfast spherical spline interpolation method (*m* = 4, *n* = 7) (Perrin et al., 1989). In average, 1.8 out of 32 channels were interpolated. The signal was then decomposed into Independent Components (extended Infomax ICA), and constant fixed-sourced artifacts were rejected using the Wavelet-ICA algorithm (Yasoda et al., 2020). Finally, data were re-referenced using the Reference Electrode Standardization Technique (Dong et al., 2017).

EEG data were epoched over a 1.5 s time window (500 ms baseline, 1 s post-stimulus), and epochs were baseline-corrected (subtraction of base vector mean) using the (−500 to 200) time range. The Linear Discriminant Analysis (LDA) Beamformer method was used to extract single-trial ERPs (Treder et al., 2016). Single-trial ERPs were extracted to be able to highlight inter-individual differences as regards emotional processing. Therefore, for each subject, LDA beamformers were divided into P200, early and late LPPs. After visual inspection, the spatial patterns were estimated as the average amplitude over trials in time windows (150–250), (400–700), and (700–1,000 ms), respectively. The covariance matrix was calculated on the full dataset. The regularization parameter γ was set at 0.5.

Inter-Trial Phase Coherence (ITPC) was computed in the 2.2–30 Hz frequency range using Morlet wavelets expanding from 1 cycle at 2.2 Hz to 2.8 cycles at 30 Hz. It was computed for each subject over trials (inter-word variability), and for each trial over subjects (inter-individual variability). Data were divided into sampling bins of 200 time points from −200 to 1,000 ms and 52 linear-spaced frequencies. The frequency analysis was oversampled with a pad ratio of 4. The bootstrap level to identify significant phase coherence relative to baseline (−500 to 200 ms) was set at *p* < 0.01. The False Discovery Rate method was implemented to correct for multiple comparisons.

Higuchi’s fractal dimension (HFD) was computed on single-trial ERPs for P200, early LPP, late LPP, and (−200, 1,000 ms) time windows separately (Selvam, 2022). HFD depends on a unique free parameter (kmax) which is the maximum precision or time scales to explore in the computation of fractal dimensionality. kmax is defined between 1 and half the number of samples of the data. HFD values increase with increasing kmax until reaching a plateau, so that the parameter is selected when HFD reaches its stationary value (Di Ieva, 2016).

#### Statistical analysis

We estimated a minimum sample size of 13 by an *a priori* power analysis for a cluster-based permutation model with four predictors: means and standard deviation for each condition [(4, 2, 3, 4, 1, 3) and 3, respectively], a minimum correlation between paired samples of 0.5, and a power of 0.9 to detect an emotion effect on single-trial ERPs for a within-subject design. The same methodology was followed for synthetization effect [mean: (6, 4, 3), SD: 3]. We followed the method and Matlab script used by Wang and Zhang (2021). Therefore, 24 utterances per emotion provided by the MESD were enough for reliable statistical analysis.

Cluster-based permutation tests were implemented to explore emotion and synthetization effects on ERP amplitude for each subject separately using Fieldtrip Toolbox (Oostenveld et al., 2011). Neurophysiological effects have spatiotemporal dimensionalities that can be used to maximize statistical sensitivity and this non-parametric approach provides information about both spatial and temporal extents of the effect, while controlling the family-wise error rate. Conditions were compared at every sample (channel × time) by means of univariate repeated measures ANOVA on a (0, 1,000 ms) time window. Samples were clustered based on spatial and temporal neighboring whose *F*-value was larger than a critical threshold (*p* < 0.05). Clusters were formed by two or more neighboring sensors. Then, cluster-level statistics were computed by the sum of *F*-values within every cluster. The maximum of cluster-level statistics was taken. To evaluate cluster-based statistics, spatiotemporal clustering was combined with non-parametric permutation analysis, with 1,000 random shuffling across conditions under the null hypothesis of data exchangeability. For each permutation, cluster-based statistics were calculated, and a distribution was built. The proportion of random partitions that resulted in a larger test statistic than the observed one was the *p*-value that was used to assess the effect. The Monte-Carlo estimate was used. When comparisons from ANOVA were significant, *post-hoc* analysis were performed by means of non-parametric cluster-based permutation dependent samples *t*-tests between each emotion. *P*-values for significance were adjusted for two-sided tests (*p* < 0.025).

Inter-individual and inter-trial dispersion of ERPs amplitude were measured by standard deviations for each subject over trials, and for each trial over subjects after rescaling the data between 0 and 1 using min-max normalization as described in Equation (3). Data rescaling was used to reduce inter-individual and inter-trial scale biases.

Then, linear effects of valence or arousal ratings on HFD extracted on P200, early LPP, late LPP, and the whole (−200, 1,000 ms) window were assessed by simple linear regression analysis. Nevertheless, residuals distribution of linear regression models outlined linearity default. Thus, non-parametric statistics were used to investigate monotonic dependencies. Correlations between HFD and behavioral responses were assessed by Spearman’s method and ρ and *p*-values were computed. Smoothing splines analysis were implemented to fit a regression model between the two variables. The smoothing parameter was optimized by means of leave-one-out cross-validation to minimize the Root-Mean-Squared Error (RMSE).

From behavioral responses, confusion matrices were computed to evaluate the qualitative emotion recognition performance for each level of voice naturalness separately. One-way repeated measures ANOVAs were performed to assess the emotion effect on valence and arousal ratings and the naturalness reduction effect on naturalness and intelligibility perceptions. Shapiro-Wilk and Mauchly’s tests were used to test for normality of residuals and sphericity, respectively. *Post hoc* comparisons were evaluated by the Tukey procedure. No violation of sphericity was highlighted. In case of non-parametricity, Friedman test was used and *post hoc* comparisons were assessed by Conover test with *p*-values adjustment by Holm method.

## Results

### Experiment 1

#### Acoustic signature of voice naturalness

**FIGURE 5:**
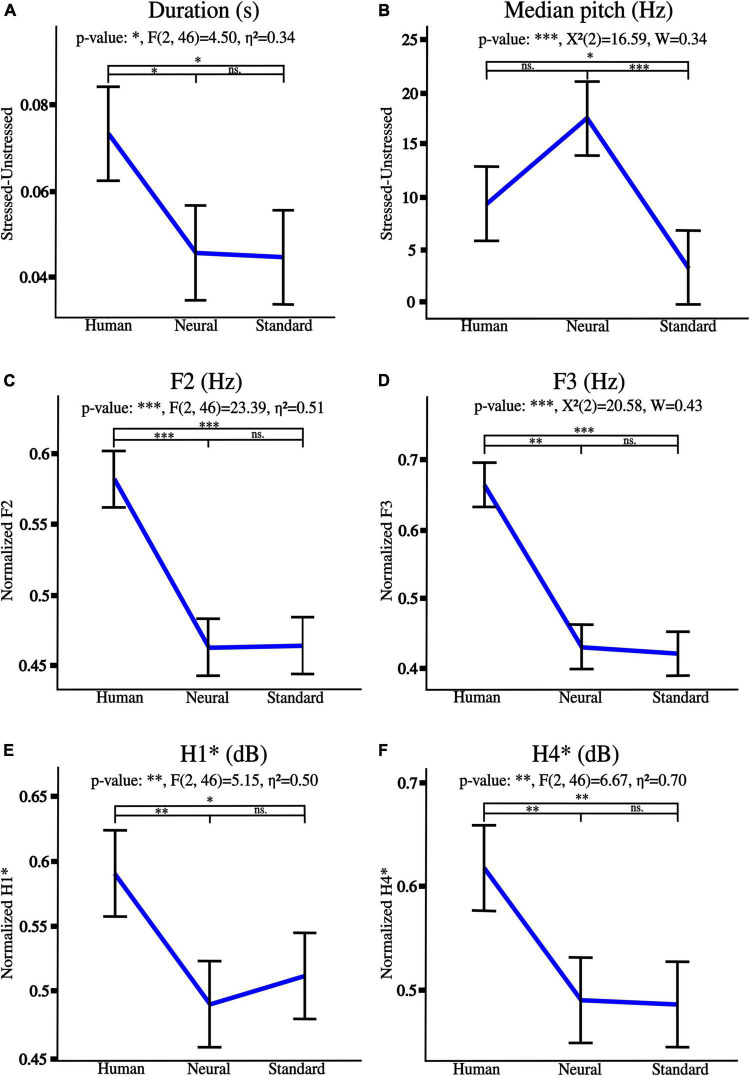
Acoustic tendencies for naturalness reduction from human to neural to standard voices as regards lexical stress: **(A)** duration, **(B)** median pitch. Graphs **(C–F)** detail spectral trends on whole utterances, respectively, for F1, F2 frequencies, H1* and H4* intensities. “*” *p* < 0.05, “^**^” *p* < 0.01, and “^***^” *p* < 0.001, ns., non-significant. η^2^ is the generalized eta-squared for ANOVA, χ^2^ is the test statistic when Friedman was applied, and W is Kendall’s effect size.

Significant trends from human to neural to standard voices are detailed in Figure 5. Note that the less natural, the least emphasized the lexical stress was as regards median pitch and duration (A, B). Besides, F2 and F3 frequencies were severely reduced (C, D), thus moving toward F1. Finally, harmonics 1 and 4 were soften by synthetization, and lower intensities were emphasized (E, F).

Besides, lexical stress tendencies were highlighted for every prosody (stressed vs. unstressed syllables of human voices utterances). Stress was significant for both duration [anger^***^: t(23) = 6.72, disgust^***^: t(23) = 4.67, fear^***^: t(23) = 5.21, happiness^**^: t(23) = 3.29, neutral^***^: *V* = 293, and sadness^***^: t(23) = 5.82], and median pitch [anger^***^: *V* = 275, disgust^**^: *V* = 43, happiness^**^ : t(23) = 3.12, and neutral^**^: *V* = 251], where “^**^” *p* < 0.01, “^***^” *p* < 0.001, V is the test statistic when Wilcoxon test was used.

Particularly, stressed syllables were longer and higher-pitched than unstressed syllables, except for unstressed syllables for disgust prosody which were higher-pitched than stressed syllables. Note that stressed and unstressed syllables of fear and sadness utterances did not differ as regards median pitch which therefore was not further edited.

#### Naturalness-reduced voices: Acoustic insight from emotional utterances

Human voice, and newly created levels 1 and 2 differed as regards lexical stress (duration and median pitch) and spectral features (F2, F3, H1*, and H4*). No significant difference between expected and observed values was highlighted for any of the edited acoustic parameters of both levels 1 and 2. See Supplementary File 1 (Supplementary Figures 1–6) for detailed general and *post hoc* statistical effects of naturalness reduction on emotional prosodies, and details about mean and standard deviations for each feature across levels. Particularly, the effect of naturalness was significant for all features measured on utterances from the six prosodies with a progressive reduction from human voice to level 1 to level 2 (*p* < 0.001; except for median pitch on happiness and neutral utterances: *p* < 0.01).

### Experiment 2

#### Event-related potentials: Emotional recognition

A significant emotion effect was observed on 21 subjects for the human voice, 17 for level 1, and 21 for level 2 (*p* < 0.05). Besides, a significant synthetization effect was highlighted on 13 subjects for anger, 14 for disgust, 12 for fear, 10 for happiness, 14 for neutral, and 13 for sadness (*p* < 0.05). Supplementary File 2 provides topography, time, sum(F), and *p*-values of significant clusters after non-parametric ANOVA for every subject for both emotion and synthetization effects.

Emotion and synthetization effects were observed with the same topography for all time windows (P200, early and late LPPs) over frontal (F3, Fz, F4), fronto-central (FC5, FC1, FC2, FC6), central (C3, Cz, C4), centro-parietal (CP5, CP1, CP2, CP6), temporal (T7, T8), and parietal (P3, Pz, P4) cortices. No inter-hemispheric lateralization was observed for any type of voice or emotion.

**FIGURE 6:**
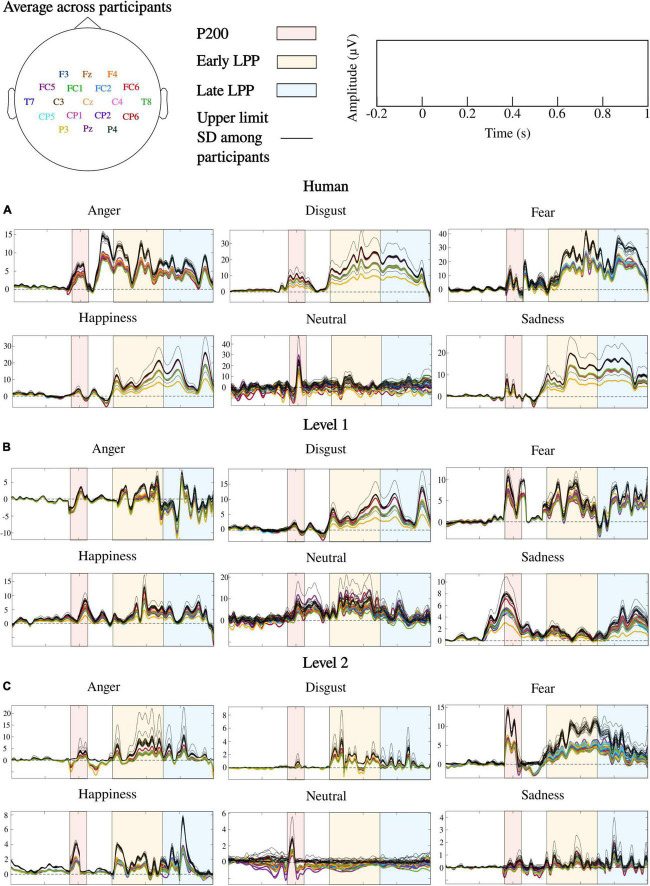
ERPs correlates of emotional processing for human and naturalness-reduced voices: **(A)** human voice, **(B)** level 1, **(C)** level 2. Colored lines are ERPs averaged over words and participants. Each color corresponds to a sensor as detailed by the topoplot in legend. Dark lines are upper limits established by standard deviations (SD) across participants. Note that frontal, fronto-central, central, centro-parietal, temporal, and parietal cortices were where emotion processing occurred during P200, early and late LPPs, and ERPs over those cortices followed similar patterns.

**FIGURE 7:**
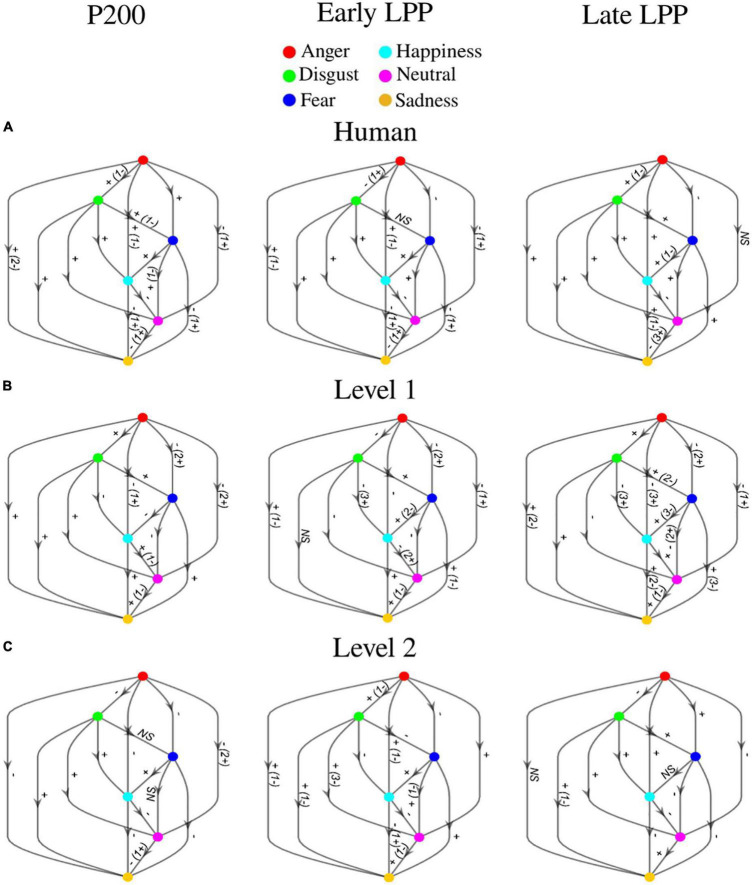
*Post hoc* comparisons for P200, early LPP, and late LPP amplitudes on **(A)** human voice, **(B)** level 1, and **(C)** level 2. Nodes represent emotions (anger: red, disgust: green, fear: dark blue, happiness: light blue, neutral: pink, sadness: orange). The direction of the arrow indicates the comparison (e.g., red toward green corresponds to anger vs. disgust). Edges labels are significant clusters sign and inform about the direction of the comparison. If the opposite sign was highlighted for a minority of subjects, it is stated between parentheses. NS, non-significant. All significant effects were located over frontal, fronto-central, central, centro-parietal, temporal, and parietal cortices.

**FIGURE 8:**
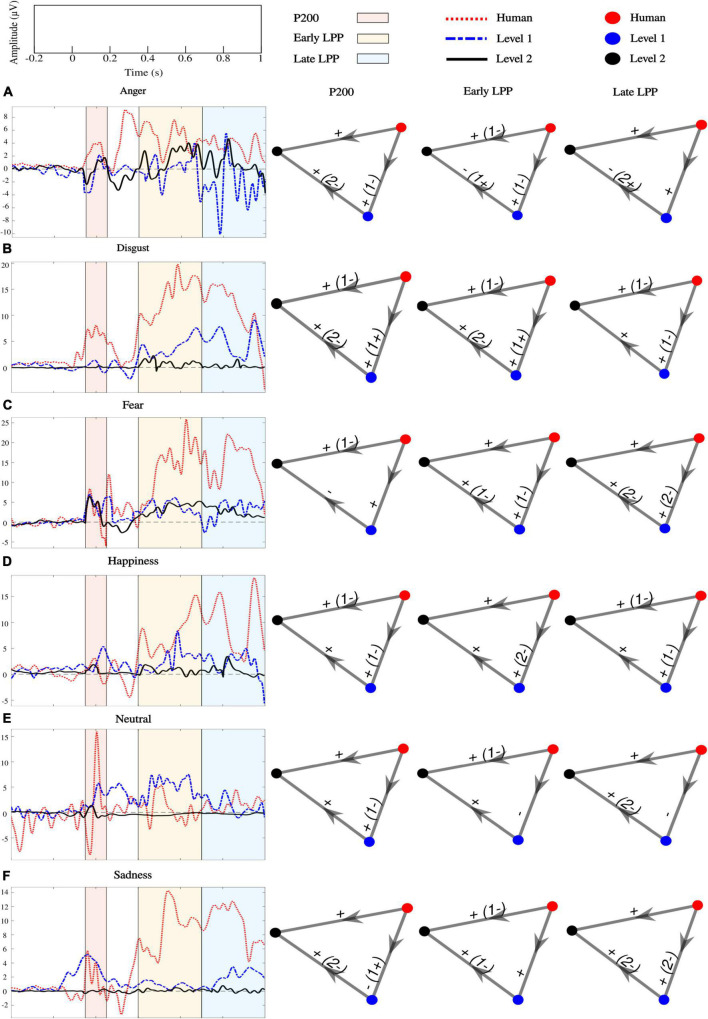
Effect of naturalness reduction on P200, early and late LPPs for **(A)** anger, **(B)** disgust, **(C)** fear, **(D)** happiness, **(E)** neutral, and **(F)** sadness processing. Colored lines are grand-average ERPs. Nodes represent levels of naturalness (human voice: red, level 1: blue, and level 2: black). The direction of the arrow indicates the comparison. Edges labels are the sign of significant clusters and inform about the direction of the comparison. If the opposite sign was highlighted for a minority of subjects, it is stated between parentheses. All clusters were located over frontal, fronto-central, central, centro-parietal, temporal, and parietal cortices.

ERPs at each sensor where emotion effect was observed, and inter-individual variability outlined by standard deviations across participants are presented in Figure 6. *Post hoc* comparisons for the whole sample of participants are detailed in Figure 7. Only significant clusters are specified (*p* < 0.025). Note that at all levels of naturalness reduction, P200 and LPP amplitudes could highlight emotion recognition (most of 2-by2 comparisons were significant). Nevertheless, the direction of the comparison may vary with naturalness reduction, highlighting differential perceptions of discrete emotions induced by synthetization. For instance, anger had higher P200 amplitude than sadness for human voice and level 1, but the opposite was observed for level 2. Particularly, the modulation of ERP responses by synthetization is presented in Figure 8. *Post-hoc* comparisons are detailed for every time window and highlight a tendency for higher amplitude at higher naturalness.

#### Event-related potentials: Inter-trial phase coherence and standard deviation

**FIGURE 9:**
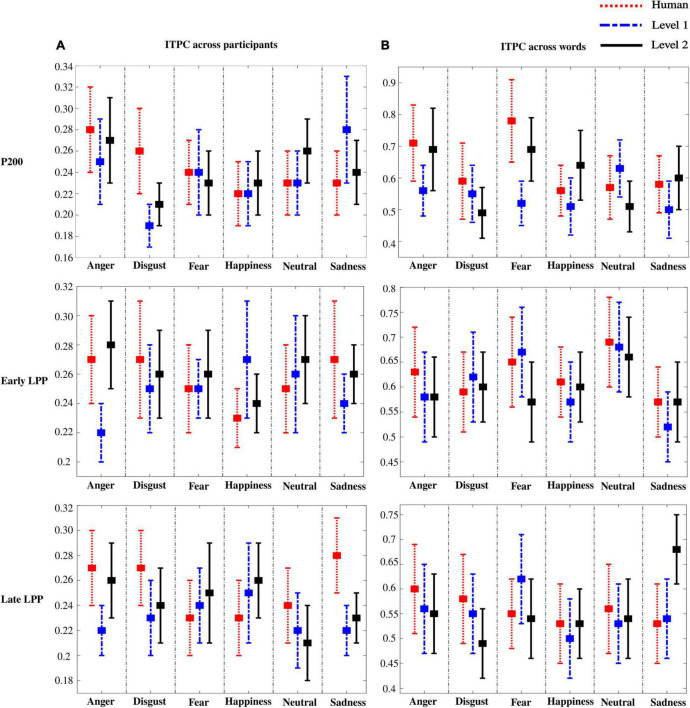
Inter-trial phase coherence (ITPC) across participants **(A)** and words **(B)**. Error bars represent SD across words **(A)** and participants **(B)**.

ITPC was computed to assess inter-individual heterogeneity in the perception of emotions. Particularly, for every word, ITPC was assessed across participants and is presented in Figure 9A. Accordingly, ITPC across words was computed to assess inter-stimulus variability for every participant and is presented in Figure 9B. Note that ITPC across participants was particularly low (∼0.25). On the contrary, across-word ITPC showed high consistency between words (∼0.6).

To confirm results from ITPC, standard deviation was computed on ERPs for every emotion across participants and across words. Similar observations were outlined: higher variability among participants (higher SD) than among words (lower SD) was observed. Results for SD are presented in Supplementary File 1 (Supplementary Figures 7, 8). Of important note, low ITPC and high SD between participants outline an individual heterogeneity that is preserved as naturalness reduces.

#### Behavioral perception of emotional prosodies

**FIGURE 10:**
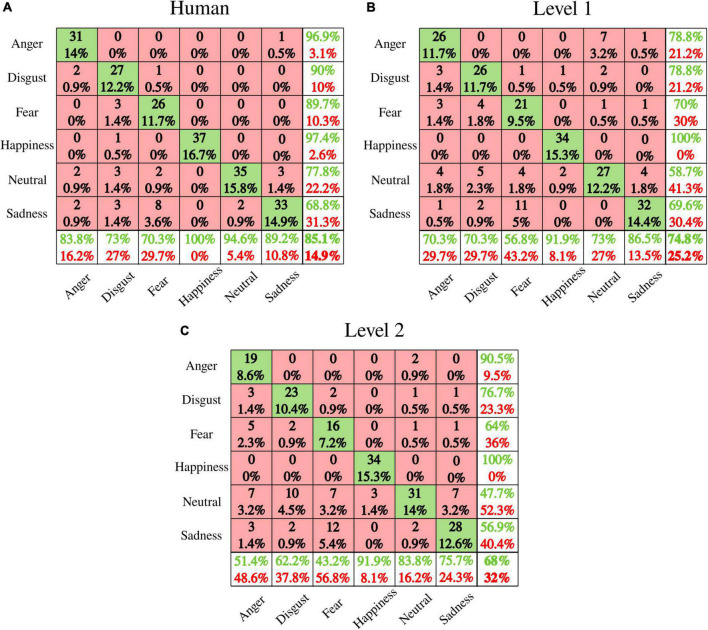
Emotion recognition performance (confusion matrices) for **(A)** human voice, **(B)** level 1, and **(C)** level 2. Rows present the predicted emotion, and columns the targeted emotion. Diagonal cells in green indicate correct recognition. Red cells correspond to emotions incorrectly recognized. Each cell includes both the number of participants that correctly/incorrectly recognized the emotion and the percentage of the total number of participants. The column on the far right states the precision (in green), and the false discovery rate (in red), both expressed in percentage. The row at the bottom of the plot details the recall (in green) and the false negative rate (in red), both expressed in percentage. The information at the bottom far right of the matrix states the overall accuracy.

**FIGURE 11:**
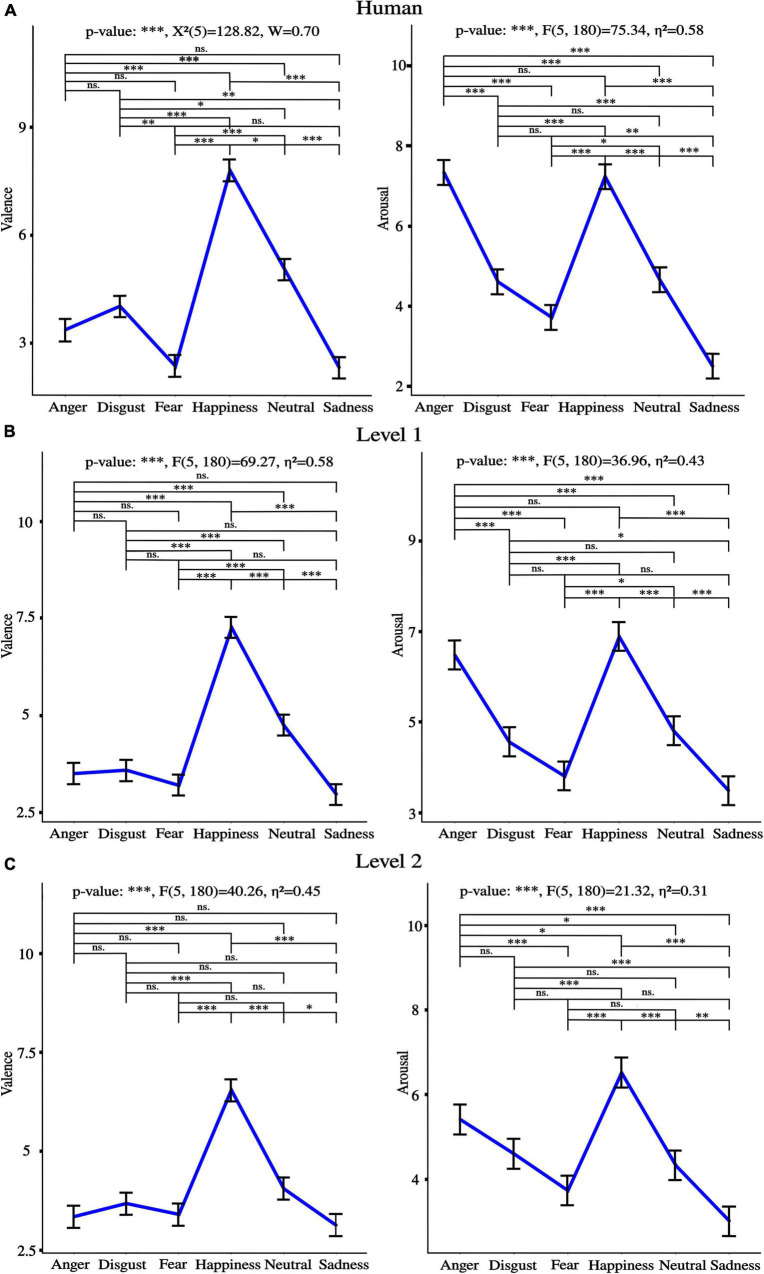
Valence and arousal ratings for **(A)** Human voice **(B)**, Level 1, and **(C)** Level 2. “*” *p* < 0.05, “^**^” *p* < 0.01, and “^***^” *p* < 0.001, ns., non-significant. η^2^ is the generalized eta-squared for ANOVA, χ^2^ is the test statistic when Friedman was applied, and W is Kendall’s effect size. Of important note, similar tendencies between emotions were observed across levels of naturalness for both valence and arousal ratings.

Performance for discrete emotions recognition as naturalness reduced is presented in Figure 10. Figure 11 shows valence and arousal ratings with results from statistical analysis for emotion effect. Results highlight lower discrete emotion recognition as naturalness reduces, however, preserved valence-arousal apprehensions.

A progressive reduction of naturalness perception was observed from human voice, to level 1 to level 2 [anger^***^: F(2, 72) = 233.59, η^2^ = 0.77, disgust^***^: F(2, 72) = 141.02, η^2^ = 0.68, fear^***^: F(2, 72) = 121.27, η^2^ = 0.60, happiness^***^: F(2, 72) = 202.45, η^2^ = 0.75, neutral^***^: χ^2^(2) = 69.18, *W* = 0.93, sadness^***^: χ^2^(2) = 51.66, *W* = 0.70], where “^***^” *p* < 0.001, χ^2^ is the test statistic when Friedman test was used, and W is Kendall’s effect size. *Post-hoc* comparisons were all significant.

The same tendency was observed for intelligibility [anger^***^; χ^2^(2) = 59.86, *W* = 0.81; disgust^***^: χ^2^(2) = 63.57, *W* = 0.86; fear^***^: F(2, 72) = 104.11, η^2^ = 0.59; happiness^***^: χ^2^(2) = 53.97, *W* = 0.73; neutral^***^: F(2, 72) = 215.15, η^2^ = 0.73; sadness^***^: χ^2^(2) = 57.74, *W* = 0.78], where “^***^” *p* < 0.001, χ^2^ is the test statistic when Friedman test was used, and W is Kendall’s effect size. *Post-hoc* comparisons were all significant.

#### Correlation between behavioral and neurophysiological data

Significant regression models and correlations were outlined as detailed in Table 2. Valence (arousal) ratings correlated negatively (positively) with HFD. Note that although ERPs time windows showed occasional correlations with valence and arousal ratings, EEG data upon the whole (−200 to 1,000 ms) window was significantly correlated with valence and arousal ratings for human, level 1, and level 2 utterances.

**TABLE 2:** Spearman correlation and regression analysis outputs between Higuchi’s fractal dimension (HFD) and valence/arousal ratings.

		P200	Early LPP	Late LPP	[−200 1000 ms]	
**Human**	ρ*; p^†^	−0.37; ***	0.01; 0.89	−0.21; ***	−0.38; **	**Valence**
	*RMSE^+^; R^2^*	5.97e-3; 0.55	4.83e-3; 0.80	5.38e-3; 0.45	3.76e-3; 0.75		
**Level 1**	*ρ; p*	0.04; 0.57	**−**0.12; 0.09	−0.48; ***	−0.59; **	
	*RMSE; R^2^*	5.92e-3; 0.90	4.55e-3; 0.91	4.67e-3; 0.89	3.75e-3; 0.91	
**Level 2**	*ρ; p*	**−**0.08; 0.27	−0.38; ***	**−**0.081; 0.27	−0.23; ***	
	*RMSE; R^2^*	5.91e-3; 0.60	4.91e-3; 0.70	4.73e-3; 0.86	4.31e-3; 0.67	
**Human**	*ρ; p*	**−**0.11; 0.14	0.11; 0.12	0.03; 0.72	0.18; **	**Arousal**
	*RMSE; R^2^*	5.98.e-3; 0.55	4.83e-3; 0.80	5.39e-3; 0.45	3.77e-3; 0.75		
**Level 1**	*ρ; p*	0.39; ***	0.27; ***	**−**0.07; 0.31	0.18; **	
	*RMSE; R^2^*	5.92e-3; 0.90	4.55e-3; 0.91	4.67e-3; 0.89	3.75e-3; 0.91	
**Level 2**	*ρ; p*	0.23; ***	**−**0.12; 0.09	0.10; 0.15	0.35; ***	
	*RMSE; R^2^*	5.92e-3; 0.60	4.91e-3; 0.70	4.73e-3; 0.86	4.31e-3; 0.67	

Significant correlations are highlighted in bold.

*ρ, Spearman’s rho; ^†^p, Spearman’s p; ^+^RMSE, Root Mean Squared Error.

**p < 0.01 and ***p < 0.001.

## Discussion

The role of naturalness reduction in emotional prosody processing has been underexplored despite the increasing use of synthesized voices in daily life areas. For the first time, we created naturalness-reduced emotional utterances and explored EEG and behavioral patterns of emotion, clarity, and naturalness understandings. The goal of this study was to clarify the human cognitive ability to decipher emotional states conveyed by synthesized voices.

### Preserved valence and arousal, but impaired discrete emotions recognition as naturalness reduces

Our behavioral data show that naturalness-reduced voices are rated similarly in the valence-arousal model as human voices. Previous studies brought to light the significance of pitch, spectral sequences, and intensity as emotional acoustic markers for valence-arousal characterization of non-human voices (Xue et al., 2018; Striepe et al., 2021). By reducing the naturalistic quality of speech, the distinctive acoustic patterns of emotional prosodies were preserved enough to guarantee correct valence and arousal apprehensions. Nevertheless, a steep reduction of discrete emotion recognition was observed from the human voice (average accuracy: 85.1%) to level 1 (74.8%) to level 2 (68%). Contrary to valence-arousal dimensionality, discrete emotion categorization involves specificity and unambiguity (Zhao et al., 2018; Kranzbühler et al., 2020). Accordingly, two emotions may share valence and/or arousal perceptions. For instance, our data highlighted disgust and fear to be both rated as “negative valence” and “low arousal.” Besides, anger and happiness shared high arousal ratings with inverse valence (respectively, negative and positive). Decreasing naturalistic cues preserved valence and arousal perceptions while making difficult the specific differentiation between emotions. The voice is indeed a rich communicative channel that helps human to express themselves through acoustic signals where speaker, lexical, and emotional acoustic dependencies may exist. For instance, data from the IEMOCAP database highlighted that emotional modulations across spectral and prosodic features account for 9.1% of the total variability while lexical and speaker modulations portray 76 and 14.9%, respectively (Mariooryad and Busso, 2014). Our study underscores the human perception of such acoustic dependencies by highlighting the concomitant reductions of discrete emotions recognition and naturalness perception. Further studies should be pursued to explore the bidirectionality of emotionality and speaker-embedded naturalness acoustic relationships. In other words, it would be relevant to ask to what extent acoustic variations that trigger emotional speech encode the perception of ecological relevance, or speaker representations capture emotional prosodies.

### Electroencephalographic time course differentially encodes discrete emotions as naturalness reduces and correlates with valence and arousal ratings

Our ERP results could be used as a starting point for addressing this issue. We report separate early differentiations of the six basic emotions captured by the P200 over human and naturalness-reduced voices. For instance, anger elicited stronger P200 than disgust when uttered by human and level 1 voices, but the inverse pattern was observed for level 2 utterances. The same observation applied for anger vs. sadness. Happy utterances from both naturalness-reduced voices triggered higher amplitude than anger, but the opposite was observed for the human voice. The same observation applied for fear vs. anger. Acoustic variabilities of naturalistic cues induced onto voice synthetization toward less ecological statistical models did not totally jeopardize emotional discriminations but triggered differential emotional salience primarily based on the integration of acoustic features. Thus, acoustic variations that encode ecological relevance directly acted on the relative emotional significance of discrete emotions. Besides, later LPP patterns followed similar trends: anger utterances induced lower early LPP and stronger late LPP than disgust when uttered with human and level 1 voices, but the opposite was observed for level 2. Fear triggered stronger late LPP than anger when uttered by level 2 but lower when uttered by human and level 1 voices. Previous research emphasized a correlational behavior between P200 and subsequent LPP amplitudes (Schirmer et al., 2013; Steber et al., 2020), which highlights the significance of early salience detection for further in-depth evaluation. Although the direct influence of P200 on LPP was not measured here, it seems that variations of acoustic cues that capture the naturalistic voice perception affected both emotional salience detection and strengthened analysis.

In line with behavioral recognition of discrete emotions, deviations of naturalness-reduced voices away from acoustic naturalistic statistical models weakened emotional judgments, specifically while listening to least ecological voices. Particularly for level 2 utterances, P200 amplitudes were not modulated by fear vs. disgust nor neutral. Furthermore, late LPP was not sensitive to anger vs. sadness acoustic variations. These observations match behavioral confusions between fear and disgust or neutral, anger and sadness for level 2 voices. Additionally, the synthetization effect highlighted on the processing of every affective prosody confirmed the fading of ERP responses with naturalness reduction. Nevertheless, although emotional integration dropped concomitantly with ecological relevance, the neuronal emotional response was never totally vanished, discrete emotional recognition was competitive at both naturalness reduction levels (74.8% overall accuracy for level 1 and 68% for level 2), and valence/arousal perceptions were preserved. Therefore, our study outlines that the potential for human-robot interactions to convey emotions by prosody (James et al., 2018) may be extended to synthesized voices.

Besides, significant correlations between valence/arousal perceptions and neurophysiological data were observed upon the whole (−200 to 1,000 ms) temporal window for both human and synthesized voices. Early and late ERPs time windows showed occasional significant correlations for either human or naturalness-reduced voices processing. Indeed, emotional speech comprehension involves linguistic and contextual apprehensions that interplay with emotional understandings. For instance, analysis of time dynamic properties of speech processing revealed that incongruencies between emotional prosody and semantics may modulate ERP responses from 100 ms after violation detection (Paulmann and Kotz, 2008) to 1,000 ms [see (Mauchand et al., 2021) for P200, N400 and (Kotz and Paulmann, 2007; Mauchand et al., 2021) for late positivity modulations]. Besides, contextual expectancies may alter word emotional understanding with stronger P200 and LPP responses when words are embedded into congruent emotional contexts (Chou et al., 2020). What is more, self-referencing contexts may enhance emotional perception [see (Herbert et al., 2011) for effect on LPP amplitude], without which negative common words may hinder source memory performance, underscored by the absence of old/new item effect on ERP amplitude between 500 and 800 ms (Pereira et al., 2021). In sum, by highlighting the relevance of the whole epoch to link neuronal processing to behavioral emotional perception, our correlational analysis may have emphasized the interactive interplay between several representational levels during online emotional speech comprehension.

### High inter-individual heterogeneity for emotion perception is preserved as naturalness reduces

Finally, we analyzed ITPC across words for each participant, and across participants for each word. ITPC is a measure of consistency of EEG spectral phase. ERPs are phase-locked responses time-locked to the stimulus. Therefore, when trial-to-trial responses follow a similar phase pattern, the ITPC should increase relative to the presentation of the stimulus (Luck and Kappenman, 2012). ITPC in combination with ERP was shown to be sensitive to speech integration in delta, theta, alpha, and beta bands between 100 and 600 ms after stimulus onset (Nash-Kille and Sharma, 2014; Sorati and Behne, 2019; Elmer et al., 2021). Our results highlight significant ITPC between words with stereotyped time-locked responses (∼0.6), and significant between-participant ITPC with partial phase synchrony (∼0.25). Low ITPC along with high ERP amplitude dispersion (SD) emphasizes miscellaneous emotional responses among subjects. Personality traits and gender may have modulated emotional responsiveness. For instance, neuroticism has been correlated with enhanced hemodynamic activity within the medial frontal cortex (Brück et al., 2011) and lower N400 when processing happy as compared with angry utterances (Ku et al., 2020). Similarly, extraversion was associated with reduced N400 while processing happy prosodies relative to anger, and both neuroticism and introversion were linked to stronger LPP responses to negative than neutral utterances (Ku et al., 2020). Elsewhere, female listeners showed larger responsivity to sadness as compared to neutral prosodies than males, reflected by stronger P200 effect (Schirmer et al., 2013). Our results reveal that reducing naturalness does not shadow subject-dependent neurophysiological responses to emotional prosodies. Nevertheless, further studies are needed to explore behavioral and neuronal relationships between personality, gender, and emotional responsiveness toward synthesized voices.

### A pioneering insight into the emotion perception of future synthesized voices

Voice naturalness was defined by its acoustic properties in Experiment 1. Then, acoustic features of human voices have been edited accordingly to create naturalness-reduced voices that match the acoustic profile of currently available synthesized voices generated by text-to-speech systems. These voices were used in Experiment 2 to assess their perception by healthy adults. As a result, behavioral scores for naturalness obtained in Experiment 2 may be generalized to the perception of synthesized voices generated by text-to-speech systems. Nevertheless, our work’s novelty relies on the emotional information still present in naturalness-reduced voices created in the present study (and lacking in text-to-speech synthesized voices). Therefore, emotional assessments indexed by psychometric scales and by neurophysiological data are specific to the perception of the newly created synthesized voices. On the other hand, the emotional perception highlighted in Experiment 2 may be a pioneering insight into the human perception of emotions conveyed by future synthesized voices that will be created by text-to-speech systems when progress will have been done to reach adequate discrete emotions induction.

### Limitations

The present study has a few limitations that should be taken into consideration. First, only the voice available in the MESD was used, and two versions of naturalness-reduced affective prosodies were created. Second, we narrowed the study to female utterances. Further research is needed to ascertain the generalization of the findings to other female voices, to male and child utterances, and to other degrees of naturalness reduction.

## Conclusion

The present study was designed to (1) explore acoustic cues of voice naturalness, (2) create naturalness-reduced synthesized versions of emotional and neutral utterances, and (3) assess behavioral and neurophysiological correlates of emotional perception conveyed by both human and synthesized voices. The results outlined acoustic dependencies between ecological relevance perception and discrete emotions recognition while valence-arousal dimensionalities proved to be unaffected by naturalistic cues variability. P200 and LPP patterns highlighted disparate time dynamics for relative emotions recognition whilst ecological relevance dropped, which was related with behavioral perceptions of both valence-arousal and discrete emotionality. Finally, ITPC and SD measurements emphasized subject-dependent time courses for processing emotional prosodies still preserved when listening to less natural voices.

Synthesized voices are nowadays embedded into our daily lives, but the neuronal integration of less naturalistic social and emotional information is still misunderstood. Further research is needed to tackle functional neuronal networks and brain dynamics associated with the emotional perception of acoustic modulations away from naturalistic models.

## Data availability statement

The original contributions presented in this study are publicly available. This data can be found here: https://data.mendeley.com/datasets/cy34mh68j9/5.

## Ethics statement

The studies involving human participants were reviewed and approved by the Ethics Committee of the School of Medicine of Tecnologico de Monterrey. The patients/participants provided their written informed consent to participate in this study. Written informed consent was obtained from the individual(s) for the publication of any potentially identifiable images or data included in this article.

## Author contributions

MD oversaw the conceptualization, validation, methodological framework development, software applications, statistical and computational analysis, data collection, providing resources, writing, reviewing, editing manuscript, and funding acquisition. LA-V and DI-Z supervised, reviewed, and edited the manuscript. All authors approved the submitted version of the manuscript.

## Footnotes

1
https://cloud.ibm.com/docs/text-to-speech?topic=text-to-speech-gettingStarted

2
https://azure.microsoft.com/en-us/services/cognitive-services/text-to-speech/#overview

3
https://www.mathworks.com/products/audio.html

4
http://www.praatvocaltoolkit.com/
