# Supplementary material for: Neuronal and behavioral affective perceptions of human and naturalness-reduced emotional prosodies
Source: Front Comput Neurosci. 2022 Nov 18;16:1022787. doi: 10.3389/fncom.2022.1022787 (PMC9716567; doi:10.3389/fncom.2022.1022787)
Supplement: Supplementary file 1 [file Data_Sheet_1.PDF]

1 Acoustic Features Extracted to Define Speech Naturalness

Supplementary Table 1. Acoustic features extracted to define speech naturalness from whole utterances.

Type	Feature	Description	Mathematical expression
Prosodic	Intensity (dB)	Mean	$\mu_2 = \sum_{n=0}^{N-1} X(n) \big/ N$ where μ_2 is the mean, N is the total number of samples and X(n) is the intensity in dB at sample n.
Voice quality	Jitter (%)	Jitter local: average absolute difference between two consecutive periods, divided by the average period.	$\left(\frac{\sum_{i=2}^{P} \lvert T_i - T_{i-1} \rvert}{(P-1)} \Big/ \frac{\sum_{i=1}^{P} T_i}{P} \right) X\ 100$ where T_i is the duration of ith period, and P is the number of periods.
		Jitter ppq5: 5-point period perturbation quotient. It is the average absolute difference between a period and the average of it and its four closest neighbors, divided by the average period.	$\left(\frac{\frac{\sum_{i=3}^{P-2} \lvert T_i - (T_{i-2} + T_{i-1} + T_i + T_{i+1} + T_{i+2} \rvert / 5}{(P-4)} \Big/ P-4}{\frac{\sum_{i=1}^{P} T_i}{P}} \right) X\ 100$ where T_i is the duration of ith period, and P is the number of periods.
	Shimmer (%)	Shimmer local: the average absolute difference between the amplitude of two consecutive periods, divided by the average amplitude.	$\left(\frac{\sum_{i=2}^{P} \lvert A_i - A_{i+1} \rvert}{(P-1)} \Big/ \frac{\sum_{i=1}^{P} A_i}{P} \right) X\ 100$ where A_i is the amplitude of ith period, and P is the number of periods.
		Shimmer rapq5: 5-point amplitude perturbation quotient. It is the average absolute difference between the amplitude of a period and the average of the amplitude of it and its four closest neighbors, divided by the average amplitude.	$\left(\frac{\frac{\sum_{i=3}^{P-2} \lvert A_i - (A_{i-2} + A_{i-1} + A_i + A_{i+1} + A_{i+2} \rvert / 5}{(P-4)} \Big/ P-4}{\frac{\sum_{i=1}^{P} A_i}{P}} \right) X\ 100$ where A_i is the amplitude of ith period, and P is the number of periods.
	Mean harmonics-to-noise ratio (HNR; dB)	Relation of the energy of harmonics against the energy of noise-like frequencies.	If 99% of the signal is composed of harmonics and 1% is noise, then HNR is defined by: $HNR = 10\log10\left(99/1\right) = 20\ dB$ Mean harmonics-to-noise ratio between time points t1 and t2 is defined by:

			$\text{HNR}=\frac{1}{(t_2-t_1)}\int_{t_1}^{t_2} dt\, x(t)$ where x(t) is the HNR (in dB) as a function of time.
	Formants (Hertz)	F1, F2, F3: Mean	
Spectral	Harmonics intensity (dB)	H1*, H2*, H3*, H4*	$H^{*} = H - \sum_{i=1}^{n} 10log_{10}\frac{(r^2+1-2r_i\cos(\omega_i))}{(r^2+1-2r_i\cos(\omega_i+\omega))(r^2+1-2r_i\cos(\omega_i-\omega))}$ where H is the amplitude of the harmonic without correction (dB), $r_i = e^{-\pi B_i}/_{F_s}$, $\omega_i = {2\pi F_i}/_{F_s}$, and $\omega = {2\pi f}/_{F_s}$, B is the bandwidth of the formant to be corrected for (Hz), F_s is the sampling frequency (Hz), F is the frequency of the formant to be corrected for (Hz), and f is the frequency of the harmonic to be corrected (Hz).

2 Naturalness-Reduced Voices: Acoustic Insight from Emotional Utterances

2.1 Anger

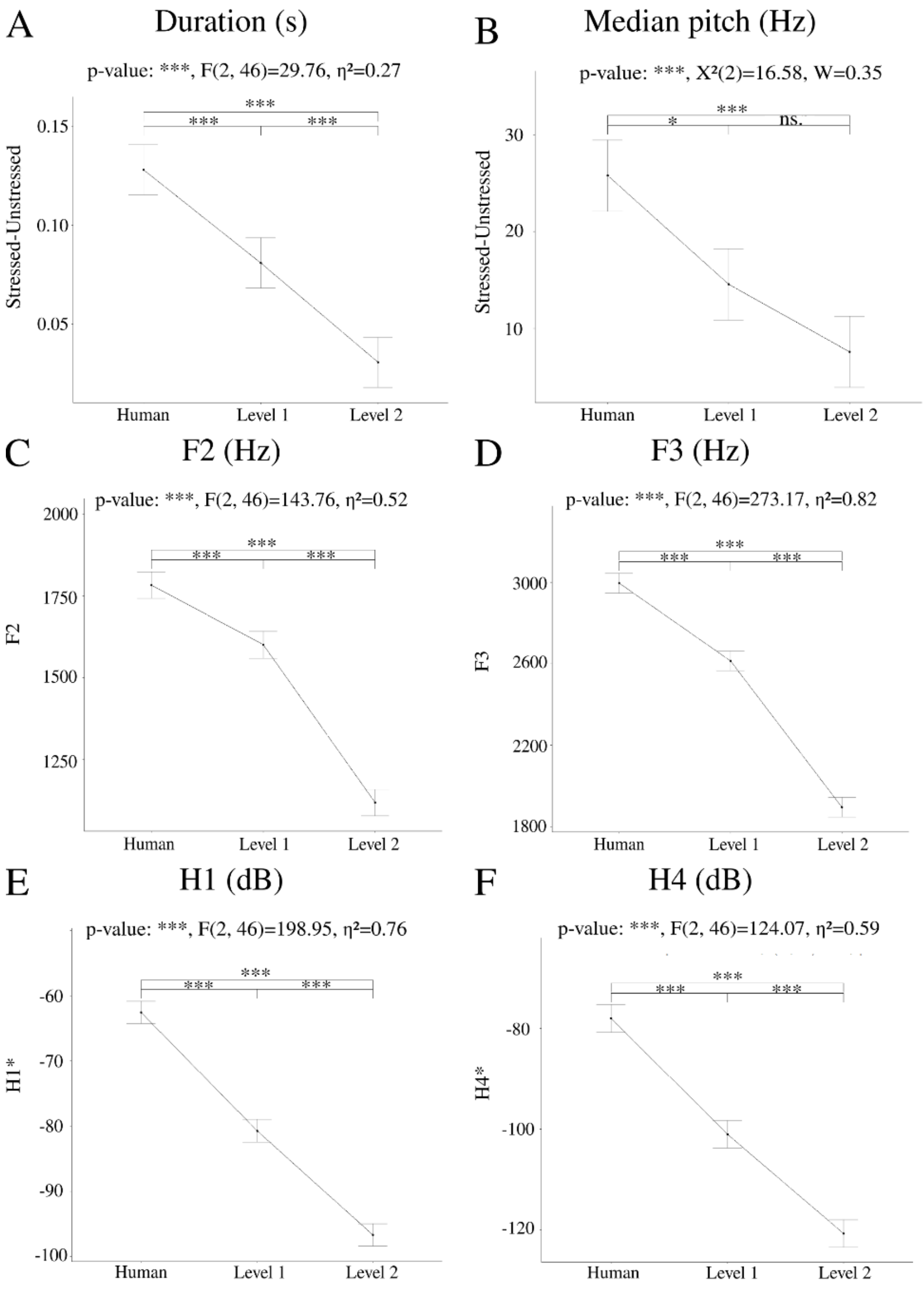

Supplementary Figure 1. Acoustic tendencies from human to level 2 of anger utterances as regards lexical stress: (A) duration, (B) median pitch. Graphs (C) to (F) detail trends on whole utterances. “ * ” $p<0.05$, “ ** ” $p<0.01$, and “ *** ” $p<0.001$, ns.: non-significant. η^2 is the generalized eta-squared for ANOVA, χ^2 is the test statistic when Friedman was applied, and W is Kendall’s effect size.

2.2 Disgust

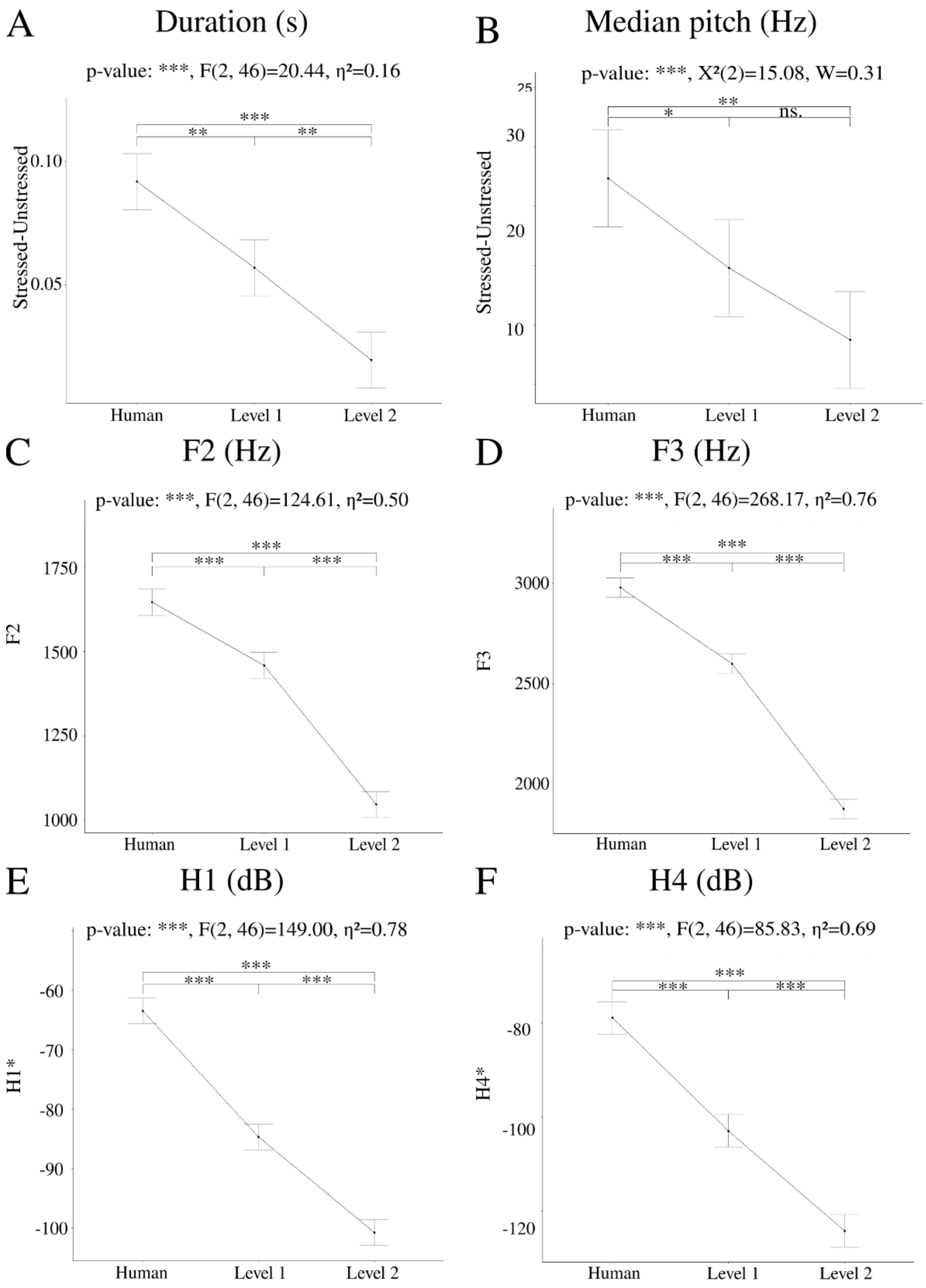

Supplementary Figure 2. Naturalness reduction on disgust emotional prosodies as regards lexical stress: (A) duration, (B) median pitch. Graphs (C) to (F) detail trends on whole utterances. " * " $p<0.05$, " ** " $p<0.01$, and " *** " $p<0.001$, ns.: non-significant. η^2 is the generalized eta-squared for ANOVA, χ^2 is the test statistic when Friedman was applied, and W is Kendall's effect size.

2.3 Fear

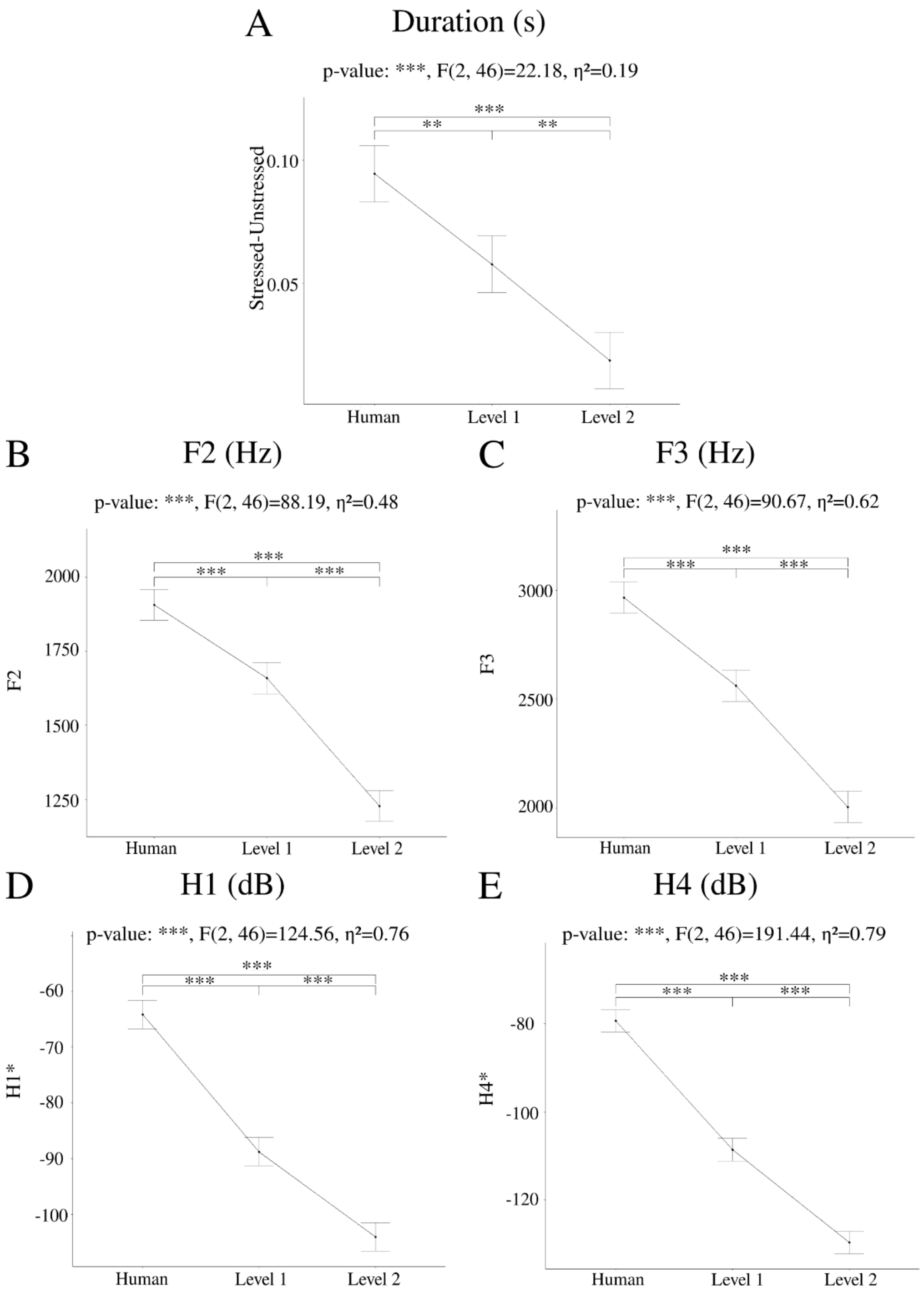

Supplementary Figure 3. Naturalness reduction on fear emotional prosodies as regards lexical stress: (A) duration. Graphs (B) to (E) detail trends on whole utterances. " * " $p<0.05$, " ** " $p<0.01$, and " *** " $p<0.001$, ns.: non-significant. η^2 is the generalized eta-squared for ANOVA, χ^2 is the test statistic when Friedman was applied, and W is Kendall's effect size.

2.4 Happiness

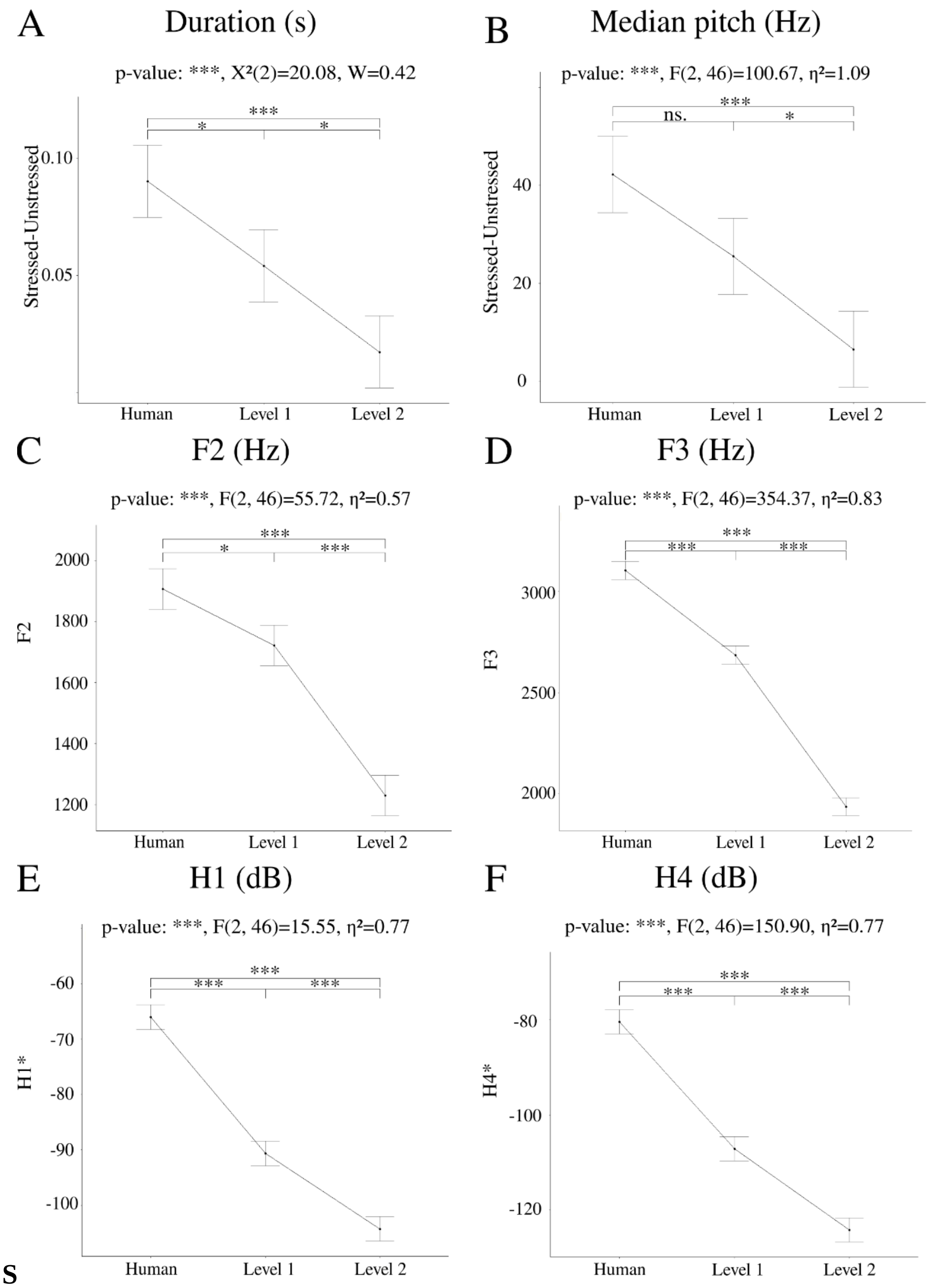

Supplementary Figure 4. Naturalness reduction on happiness emotional prosodies as regards lexical stress: (A) duration, (B) median pitch. Graphs (C) to (F) detail trends on whole utterances. “ * ” $p<0.05$, “ ** ” $p<0.01$, and “ *** ” $p<0.001$, ns.: non-significant. η^2 is the generalized eta-squared for ANOVA, χ^2 is the test statistic when Friedman was applied, and W is Kendall’s effect size.

2.5 Neutral

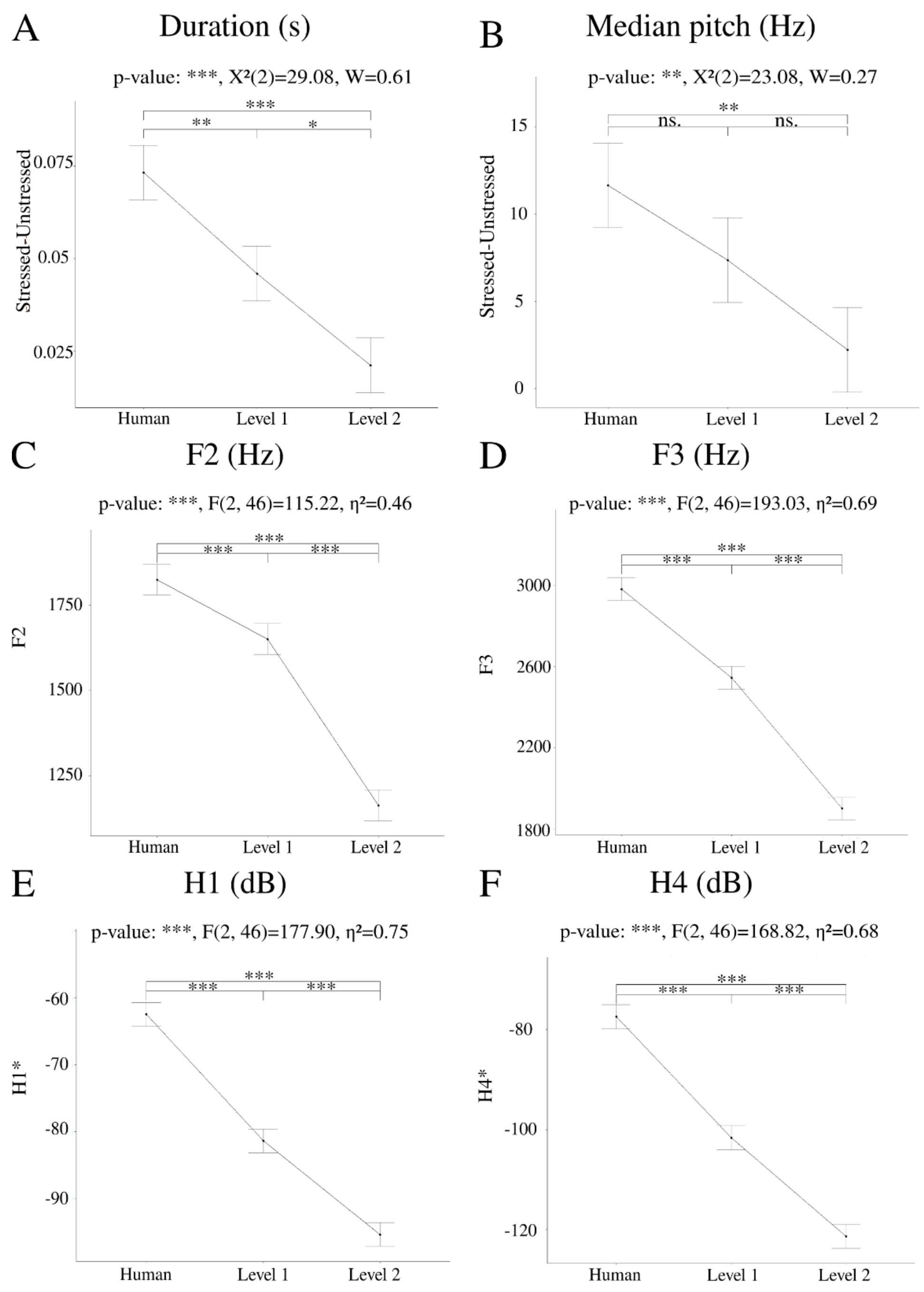

Supplementary Figure 5. Naturalness reduction on neutral emotional prosodies as regards lexical stress: (A) duration, (B) median pitch. Graphs (C) to (F) detail trends on whole utterances. " * " $p<0.05$, " ** " $p<0.01$, and " *** " $p<0.001$, ns.: non-significant. η^2 is the generalized eta-squared for ANOVA, χ^2 is the test statistic when Friedman was applied, and W is Kendall's effect size.

2.6 Sadness

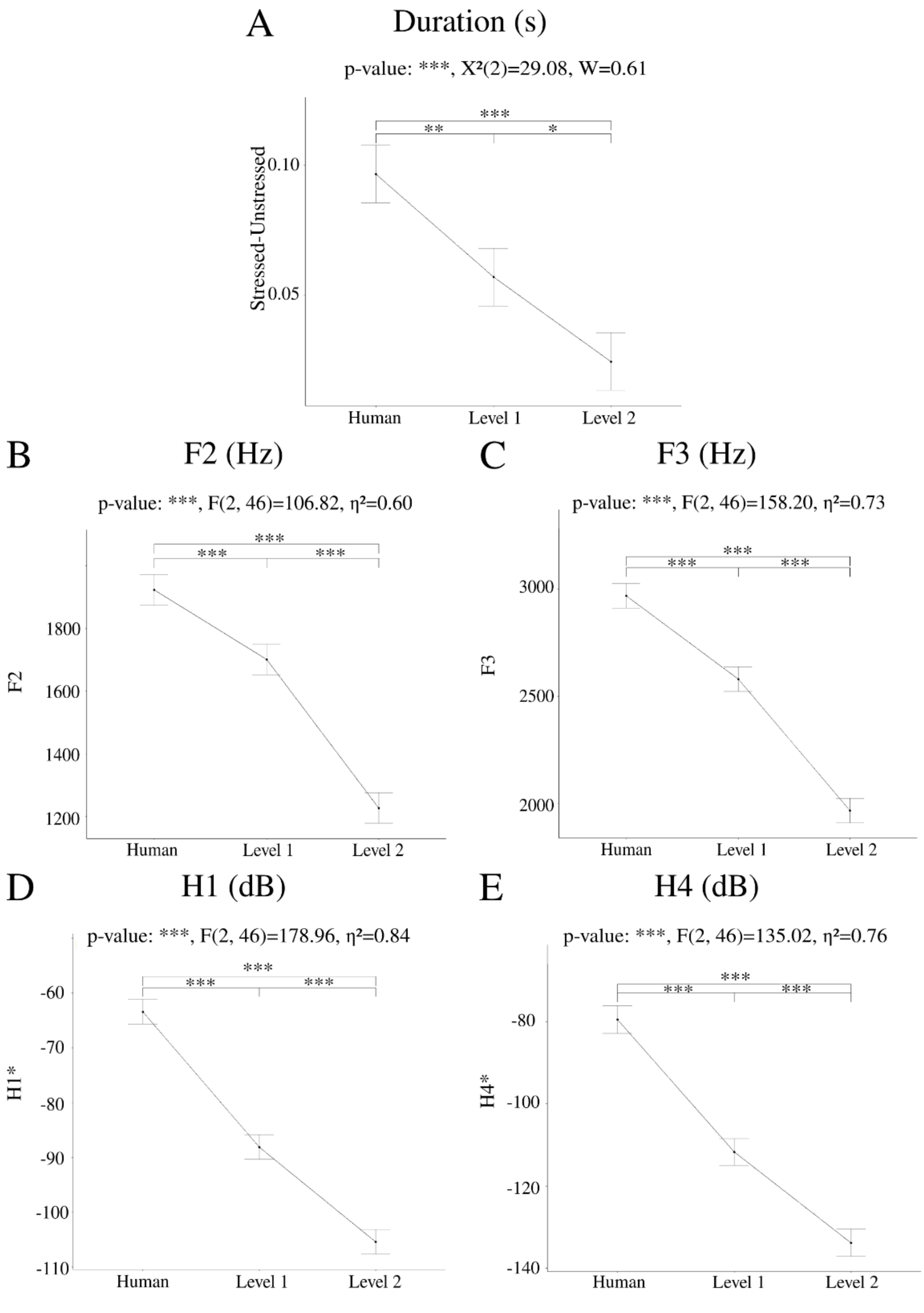

Supplementary Figure 6. Naturalness reduction on sadness emotional prosodies as regards lexical stress: (A) duration. Graphs (B) to (E) detail trends on whole utterances. " * " $p<0.05$, " ** " $p<0.01$, and " *** " $p<0.001$, ns.: non-significant. η^2 is the generalized eta-squared for ANOVA, χ^2 is the test statistic when Friedman was applied, and W is Kendall's effect size.

3 ERP: Standard Deviation

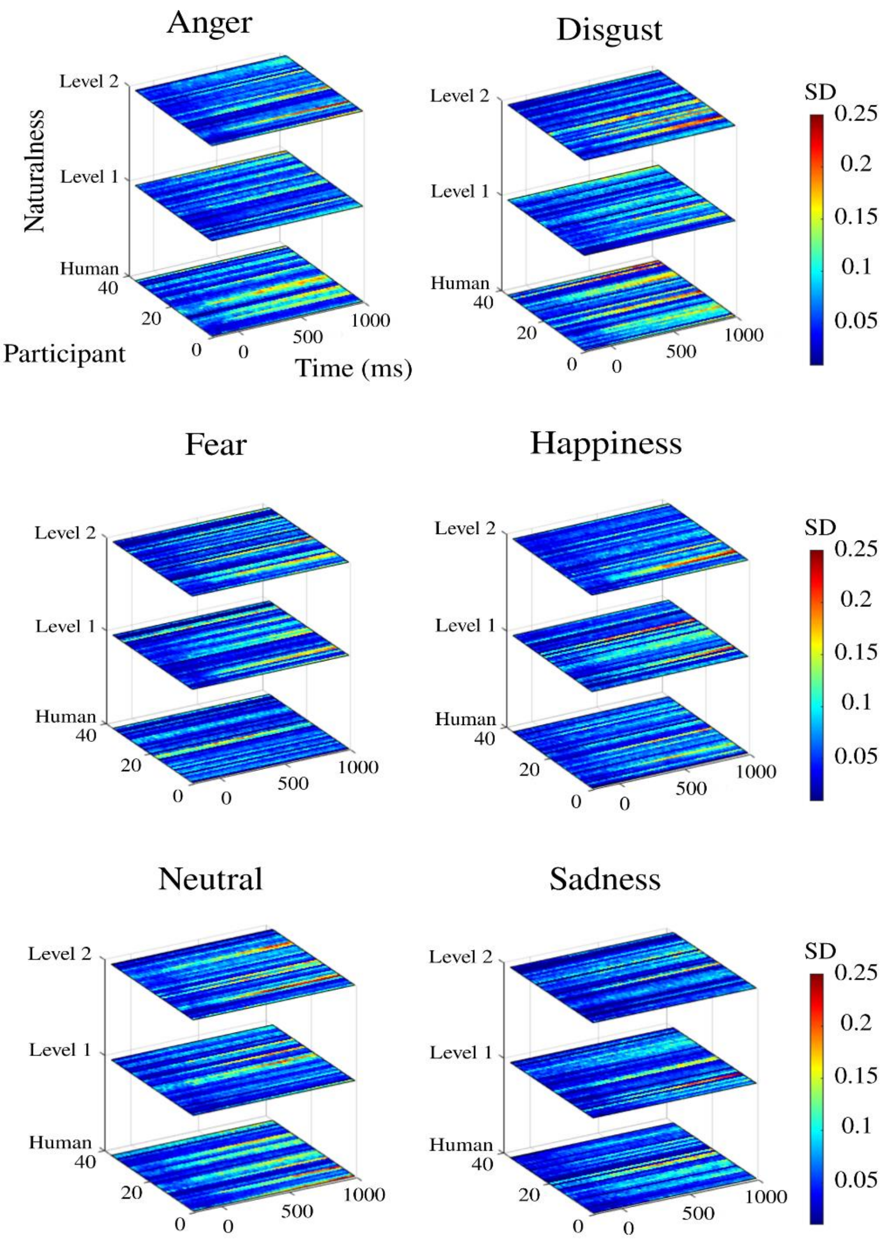

Supplementary Figure 7. Standard deviation of ERPs across participants.

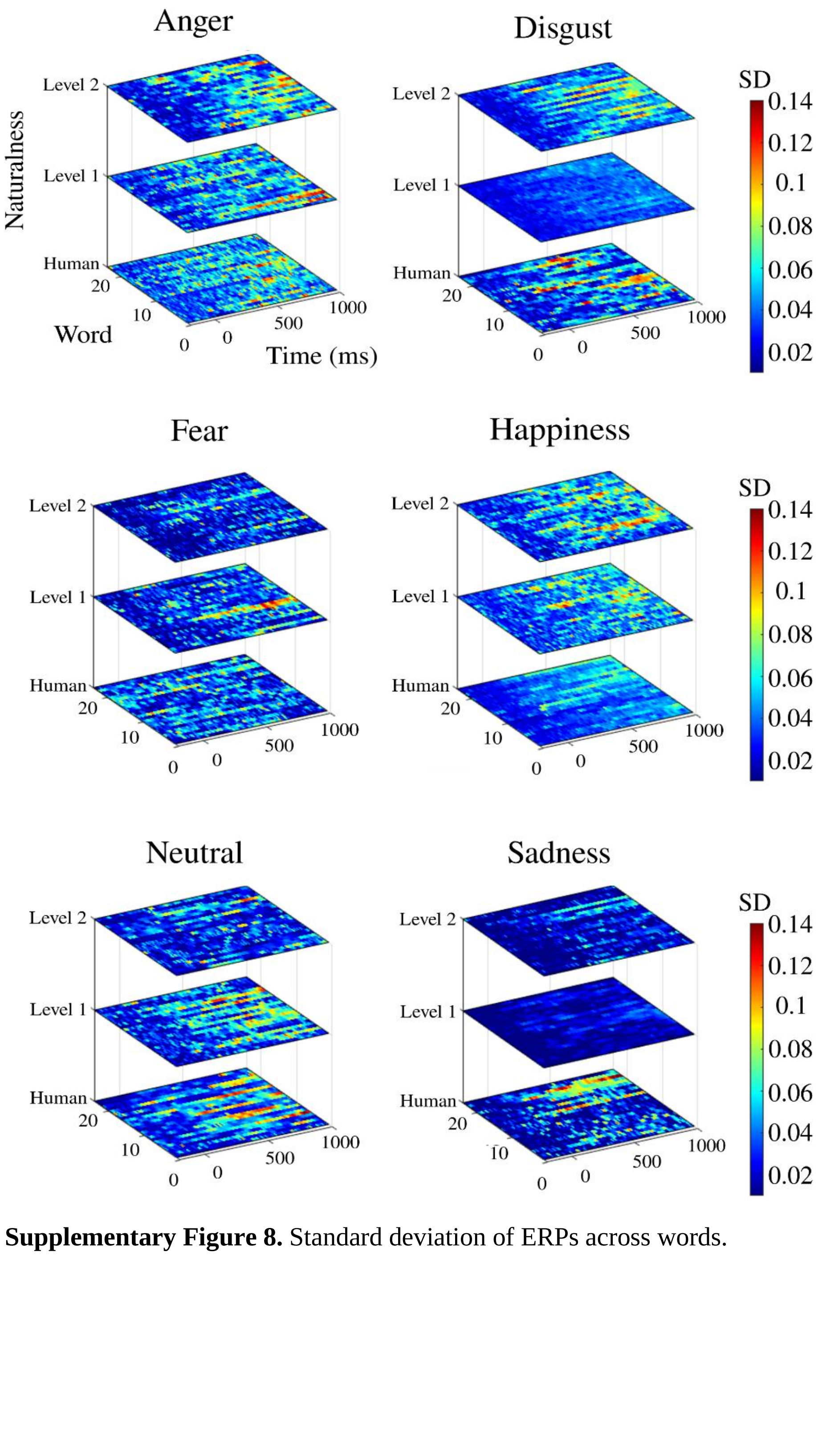

Supplementary Figure 8. Standard deviation of ERPs across words.
